# Oligodendrocyte‐Targeted SGK1.1 Inhibition Alleviates White Matter Injury and Cognitive Impairment in Alzheimer's Disease via Modulation of HDAC2 Phosphorylation

**DOI:** 10.1002/cns.71150

**Published:** 2026-09-08

**Authors:** Shiji Deng, Shenghan Gao, Chao Zhou, Zhen Lan, Huiya Li, Jiang Chen, Yuhao Xu, Jiashuo Li, Pinyi Liu, Siyuan Zhang, Xinyu Bao, Tingting Wang, Yun Xu, Xiaolei Zhu

**Affiliations:** ^1^ Department of Neurology Nanjing Drum Tower Hospital, Affiliated Hospital of Medical School, Nanjing University Nanjing China; ^2^ The State Key Laboratory of Pharmaceutical Biotechnology, Division of Immunology, Medical School Nanjing University Nanjing China; ^3^ Department of Neurology, Nanjing Drum Tower Hospital Chinese Academy of Medical Sciences & Peking Union Medical College Nanjing Jiangsu China; ^4^ MOE Key Laboratory of Model Animal for Disease Study Nanjing University Nanjing China; ^5^ State Key Laboratory of Pharmaceutical Biotechnology and Institute of Translational Medicine for Brain Critical Diseases Nanjing University Nanjing China; ^6^ Jiangsu Key Laboratory for Molecular Medicine, Medical School Nanjing University Nanjing China; ^7^ Nanjing Neurology Clinical Medical Centre, and Centre for Central Nervous Disease and Neuroscience Nanjing China; ^8^ Nanjing Key Laboratory for Cardiovascular Information and Health Engineering Medicine Nanjing China

**Keywords:** Alzheimer's disease, HDAC2, oligodendrocyte, SGK1.1, white matter

## Abstract

**Background:**

White matter (WM) abnormalities contribute to cognitive decline in Alzheimer's disease (AD). However, the mechanisms underlying AD‐associated WM vulnerability remain incompletely understood, and effective therapeutic strategies to improve WM pathology are still limited.

**Methods:**

*Sgk1.1*‐specific RNAscope combined with Olig2 immunostaining was used to assess the cellular distribution of *Sgk1.1* transcripts. Oligodendrocyte‐targeted adeno‐associated virus (AAV)‐mediated *Sgk1.1* knockdown was performed in 5 × FAD mice. *Sgk1.1* overexpression was conducted in primary oligodendrocyte precursor cells (OPCs) in vitro. Phosphoproteomic profiling, co‐immunoprecipitation, Phos‐tag Western blot, and phospho‐site mutagenesis were used to identify downstream mechanisms. A bilateral common carotid artery stenosis (BCAS) model was employed to assess broader relevance.

**Results:**

*Sgk1.1* mRNA was predominantly elevated in oligodendrocyte‐lineage cells of 5 × FAD mice prior to overt myelin loss. Targeted knockdown of oligodendroglial *Sgk1.1* ameliorated cognitive deficits, preserved myelin ultrastructure, and promoted oligodendrocyte maturation. Conversely, *Sgk1.1* overexpression arrested OPC differentiation and suppressed myelin gene expression in vitro. Mechanistically, SGK1.1 promoted HDAC2 phosphorylation at S422/S424, and blocking this phosphorylation partially reversed SGK1.1‐induced maturation deficits. Furthermore, oligodendrocyte‐targeted *Sgk1.1* overexpression exacerbated WM injury and cognitive impairment in the BCAS model.

**Conclusion:**

Our findings identify oligodendrocyte‐targeted SGK1.1 inhibition as a tractable therapeutic strategy to preserve WM integrity and cognitive function, and define HDAC2 S422/S424 phosphorylation as a mechanistic node linking SGK1.1 signaling to impaired myelination in AD and related demyelinating disorders.

## Graphical abstract

We found an early *Sgk1.1* mRNA upregulation within oligodendrocyte‐lineage cells of 5 × FAD mice, implicating SGK1.1 signaling as an early driver of myelin pathology. Mechanistically, SGK1.1 promotes HDAC2 phosphorylation at S422/S424, thereby impairing oligodendrocyte maturation. Conversely, oligodendrocyte‐targeted *Sgk1.1* knockdown ameliorates myelin deficits and cognitive impairment.

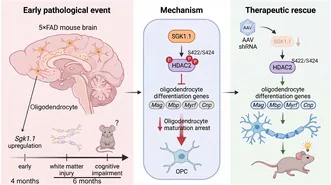

**Abbreviations**
AAV: adeno‐associated virus
AD: Alzheimer's disease
BCAS: bilateral common carotid artery stenosis
HDAC2: histone deacetylase 2
MCI: mild cognitive impairment
OPCs: oligodendrocyte precursor cells
PBS: phosphate‐buffered saline
qRT–PCR: quantitative real‐time PCR
SGK1: serum‐ and glucocorticoid‐regulated kinase 1
TEM: transmission electron microscopy
WM: white matter
WT: wild‐type

## 1 Introduction

Alzheimer's disease (AD) is the leading cause of dementia and is characterized by progressive impairments in memory and other cognitive domains [1, 2, 3]. While amyloid‐β plaques and tau neurofibrillary tangles remain central pathological hallmarks, increasing evidence indicates that these features alone do not fully account for disease progression or the severity of cognitive decline [4, 5, 6, 7]. In recent years, converging data from neuroimaging, neuropathological analyses, and single‐cell transcriptomic studies have highlighted white matter (WM) abnormalities and oligodendrocyte lineage dysfunction as prominent features of AD that may arise early during disease progression and correlate closely with cognitive impairment [8, 9, 10, 11, 12, 13]. However, the mechanisms mediating AD‐associated demyelination—particularly oligodendrocyte‐intrinsic drivers that impair myelination and cognition—remain insufficiently defined.

WM integrity and myelin are critical for cognitive function, and disruption of myelin can impair learning and memory even without overt neuronal loss [14, 15, 16]. Myelin formation and lifelong maintenance depend on the maturation of oligodendrocyte lineage cells [17, 18, 19, 20], and impaired oligodendrocyte maturation compromises myelin production and axonal support, increasing WM vulnerability and contributing to cognitive dysfunction [21, 22, 23]. Consistent with this view, WM and oligodendrocyte lineage abnormalities in AD are increasingly recognized as active contributors to disease pathophysiology rather than late secondary changes [11, 24, 25]. Recent studies show that corpus callosum—the largest and most myelin‐rich commissural tract—exhibits early structural and microstructural abnormalities in AD that correlate with cognitive decline, while WM alterations are also detected in cortical and hippocampal regions relevant to memory networks [8, 26, 27, 28].

At the molecular level, oligodendrocyte maturation is governed by coordinated transcriptional and epigenetic programs [29, 30]. Histone deacetylases HDAC1 and HDAC2 act as key gatekeepers of oligodendrocyte lineage progression, and genetic/functional studies demonstrate that HDAC1/2 are required for oligodendrocyte differentiation [31, 32]. Importantly, HDAC activity is dynamically regulated by post‐translational modifications, including phosphorylation, which can influence enzymatic activity, subcellular localization, and protein–protein interactions [33, 34]. Despite the established role of HDAC2 in oligodendrocyte maturation, whether phosphorylation‐dependent regulation of HDAC2 serves as a signaling relay that links upstream pathways to oligodendrocyte maturation failure and WM vulnerability in AD remains incompletely understood.

Serum‐ and glucocorticoid‐regulated kinase 1 (SGK1) is an AGC kinase that integrates cellular stress, metabolic, and survival signaling, and has been implicated in neuronal excitability, synaptic function, and stress responses in the central nervous system [35, 36, 37, 38]. More recently, SGK1 has been linked to AD‐relevant processes, including neuronal dysfunction and tau‐associated pathology, with elevated SGK1 expression reported in vulnerable neuronal populations in AD brains [39, 40]. In addition to neuronal contexts, emerging evidence suggests that SGK1 signaling may participate in myelin biology. Previous studies have shown that *Sgk1* is dynamically regulated in oligodendrocytes and that SGK1 signaling can influence oligodendrocyte differentiation and myelination [41, 42, 43, 44]. Importantly, these studies did not resolve SGK1.1 separately. A brain‐enriched splice isoform, SGK1.1, exhibits distinct subcellular targeting and regulatory properties compared with canonical SGK1, suggesting potentially distinct regulatory roles within the nervous system [45]. Nevertheless, whether SGK1.1 operates within oligodendrocytes to regulate maturation and myelin integrity in the adult brain, and whether such mechanisms contribute to WM vulnerability and cognitive impairment in AD, has not been explored.

Here, we identified oligodendrocyte‐intrinsic SGK1.1 as an early and causal regulator of WM vulnerability in AD models. We showed that an early, oligodendrocyte lineage‐associated increase in *Sgk1.1* mRNA preceded overt myelin loss in 5 × FAD mice and that oligodendrocyte‐targeted *Sgk1.1* knockdown preserved myelin ultrastructure, restored oligodendrocyte maturation, and improved cognitive performance. Mechanistically, phosphoproteomic profiling and functional validation converged on HDAC2 phosphorylation at S422/S424 as a prominent SGK1.1‐responsive event, and mutation of these sites partially reversed SGK1.1‐driven oligodendrocyte maturation arrest. Finally, we further examined SGK1.1 function in a distinct white matter injury model and found that oligodendrocyte lineage–targeted *Sgk1.1* overexpression aggravated demyelination and cognitive deficits in a vascular demyelination model. Together, our findings establish SGK1.1 as a regulator of oligodendrocyte maturation and white matter integrity, highlighting its potential as a therapeutic target for AD and demyelinating disease.

## 2 Materials and Methods

### 2.1 Animals

All animals were group‐housed under a controlled 12 h light/dark cycle (lights on at 8:00 a.m.) with free access to food and water. Breeding pairs of 5 × FAD mice (Cat. No. NM‐KI‐233916) and WT mice were obtained from Shanghai Model Organisms Center Inc. All experimental 5 × FAD mice and littermate wild‐type (WT) controls used in this study were generated from this in‐house colony. All in vivo experiments in the present study were conducted in male mice. Eight‐week‐old male C57BL/6J mice (20–22 g) used for BCAS surgery were purchased from the Model Animal Research Center of Nanjing University (Nanjing, Jiangsu, China). All animal procedures were reviewed and approved by the Institutional Animal Care and Use Committee of Nanjing University (2025AE01031).

### 2.2 Bilateral Common Carotid Artery Stenosis (BCAS) Model

The BCAS procedure was performed according to a previously published protocol [46]. Briefly, mice were anesthetized with 2% isoflurane delivered in 33% oxygen, while core temperature was kept at 37.0°C ± 0.5°C using a temperature‐controlled heating system. A midline ventral neck incision was made to access the carotid region, and the common carotid arteries (CCAs) were identified and freed from the surrounding carotid sheath. The right CCA was then gently elevated and guided through the turns of a microcoil (inner diameter 0.18 mm, pitch 0.50 mm, total length 2.5 mm; Sawane Spring Co., Japan), after which the coil was carefully placed around the vessel. The same manipulation was subsequently carried out on the left CCA. Sham‐operated animals underwent the same anesthesia and surgical exposure but did not receive microcoil placement.

### 2.3 Stereotaxic Viral Delivery

For oligodendrocyte‐targeted knockdown of *Sgk1.1*, 4‐month‐old 5 × FAD mice and their littermate WT controls were first acclimated to the stereotaxic surgery room for 10 consecutive days. For surgery, mice were anesthetized and positioned in a stereotaxic apparatus (RWD, China). The scalp was incised and the skull exposed, and small burr holes were drilled at the designated coordinates. Viral injections were performed using a glass micropipette connected to a Hamilton microsyringe driven by a Micro 4 pump (WPI, USA), delivering virus at a constant rate over 10 min per site. To minimize backflow, the injection needle was kept in place for an additional 10 min before withdrawal.

For shRNA‐mediated knockdown experiments, the Adeno‐associated virus (AAV), AAV‐MBP‐EGFP‐5′miR‐30a‐shRNA1(Sgk1.1)‐3′‐miR30a‐WPREs (AAV‐MBP‐EGFP‐shRNA1(Sgk1.1), 5.71 × 10^12^ vg mL^−1^), was bilaterally injected into the cortex (bregma −2.03 mm, lateral ±1.25 mm, depth 0.75 mm), hippocampus (bregma −2.03 mm, lateral ±1.25 mm, depth 2.00 mm), and corpus callosum (bregma +2.03 mm, lateral ±1.25 mm, depth 1.25 mm), with a volume of 400 nL per site. Control‐treated 5 × FAD mice and WT littermates received equal volumes of the corresponding control virus (AAV‐MBP‐EGFP, 5.13 × 10^12^ vg mL^−1^) at identical stereotaxic coordinates.

For phosphoproteomic analysis in 4‐month‐old 5 × FAD mice and BCAS experiments in 2‐month‐old C57BL/6J mice, AAV‐MBP‐3 × FLAG‐Sgk1.1‐P2A‐EGFP‐WPREs (AAV‐MBP‐3 × FLAG‐Sgk1.1‐EGFP, 7.13 × 10^12^ vg mL^−1^) was delivered bilaterally into the corpus callosum at a volume of 500 nL per side, while control animals received matched control virus (AAV‐MBP‐EGFP‐WPRE‐hGH polyA, 2.50 × 10^12^ vg mL^−1^) injections using the same procedure.

### 2.4 Behavioral Experiments

All behavioral procedures were performed during the light phase by investigators blinded to group allocation. To minimize stress‐related variability, mice were gently handled for 3 consecutive days before the first test.

Morris water maze (MWM): Spatial learning and memory were assessed using the Morris water maze. The paradigm consisted of a 6‐day schedule with two stages. During the acquisition period (Days 1–5), mice were trained to locate a hidden escape platform in a circular pool (120 cm in diameter; 40 cm in height) filled with water to a depth of 25 cm. The platform (6 cm diameter) was positioned in a fixed location and submerged below the water surface. Each mouse completed four trials per day (maximum 60 s per trial). The mean escape latency across the four trials was used as the daily learning index. Specifically, escape latency on Day 5 of the training phase was used for group comparisons. On Day 6 (probe test), the platform was removed and mice were allowed to swim freely for 60 s. Probe outcomes, including swimming speed, number of platform crossings, time spent in the target quadrant, and latency to first entry into the target quadrant, were recorded and quantified using ANY‐maze software (Stoelting, USA).

Y‐maze: Short‐term working memory was evaluated using a Y‐maze composed of three identical arms (40 cm long × 10 cm wide × 12 cm high) arranged at 120° relative to each other. Mice were placed in the maze and permitted to explore freely for 5–10 min. Arm entries were recorded, and spontaneous alternation was calculated as the proportion of overlapping triplets in which the animal entered all three arms consecutively relative to the total number of possible alternations. The apparatus was wiped with 75% ethanol between animals to remove olfactory traces.

Open field test (OFT): General locomotion and anxiety‐like behavior were examined in the open field test. Each mouse was placed in the center of a square arena (40 × 40 × 50 cm) and allowed to explore for 10 min while a camera mounted above the chamber recorded movement trajectories. Total distance traveled and time spent in center versus corner regions were extracted and analyzed using ANY‐maze (Stoelting).

### 2.5 RNAscope In Situ Hybridization and Immunofluorescence Co‐Detection

RNAscope in situ hybridization was performed in combination with Olig2 immunofluorescence staining with reference to a previously described RNA–protein co‐detection procedure [47]. A custom RNAscope probe targeting mouse *Sgk1.1* was designed in consultation with ACD/Bio‐Techne based on the RefSeq transcript NM_001161845.2 and directed against an SGK1.1‐distinguishing sequence region. Brain cryosections were mounted on adhesion microscope slides and processed using the RNAscope Multichannel Fluorescence assay and RNA–Protein Co‐Detection reagents (ACD/Bio‐Techne, USA) according to the manufacturer's instructions.

Briefly, sections were post‐fixed in 4% paraformaldehyde, dehydrated through graded ethanol, treated with hydrogen peroxide, and permeabilized with 0.1% Tween‐20. After blocking with 2% BSA, sections were incubated overnight at 4°C with an anti‐Olig2 antibody (Calbiochem, USA, OP80). Sections were subsequently re‐fixed in 4% paraformaldehyde and treated with Protease Plus at 40°C, followed by hybridization with the custom *Sgk1.1* probe for 2 h at 40°C. RNAscope signals were amplified according to the manufacturer's protocol and visualized using TSA‐based fluorophore detection. Olig2 immunoreactivity was detected with an appropriate fluorophore‐conjugated secondary antibody, and nuclei were counterstained with DAPI.

Images were acquired using an Olympus FV3000 confocal microscope. The *Sgk1.1*‐positive area associated with the Olig2‐positive and Olig2‐negative cellular compartments was quantified using ImageJ and expressed relative to the corresponding cell number. For age‐course comparisons, values were normalized to the mean value of Olig2‐positive cells from 4‐month‐old WT mice; for the knockdown experiment, values were normalized to the corresponding 5 × FAD‐GFP control group.

### 2.6 Immunofluorescence Staining

Mice were deeply anesthetized and transcardially perfused with 0.9% saline, followed by fixation with 4% paraformaldehyde. Brains were cryoprotected in sequential 15% and 30% sucrose solutions for 24 h each, and sectioned into 30 μm coronal slices using a cryostat microtome (Leica, Wetzlar, Germany).

For immunofluorescence, sections were rehydrated, permeabilized with 0.1% Triton X‐100 for 20 min, and blocked in 2% BSA for 2 h at room temperature. Sections were incubated overnight at 4°C with primary antibodies against MAG (Abcam, USA, ab89780), MBP (Abcam, USA, ab7349), APC‐CC1 (Millipore, USA, ABN899), Olig2 (Calbiochem, USA, OP80), and PDGFRα (CST, USA, 3174S). The next day, sections were incubated with appropriate fluorophore‐conjugated secondary antibodies (Invitrogen, USA) for 1.5 h at room temperature, protected from light. Images were acquired using a confocal microscope (Olympus FV3000, Japan) and analyzed with ImageJ software (Version 1.5, NIH, USA).

### 2.7 Black‐Gold Staining

Black‐Gold staining was performed using a Black‐Gold II Myelin Staining Kit (Biosensis, USA) to evaluate myelin distribution in the BCAS model according to the manufacturer's protocol. Briefly, 0.3% Black‐Gold II and 1% sodium thiosulfate solutions were warmed to 65°C. Coronal brain sections were rehydrated and then immersed in the Black‐Gold II working solution at 65°C for approximately 12 min, until myelinated fibers appeared dark red. Sections were rinsed with distilled water and subsequently differentiated in sodium thiosulfate solution at 65°C for 3 min. After another distilled‐water rinse, sections were dehydrated through graded ethanol, cleared in xylene for 2 min, and mounted with neutral balsam. Brightfield images were acquired using a light microscope (Olympus BX51, Japan) and quantified with ImageJ.

### 2.8 Western Blotting

Western blotting was carried out following standard procedures [46]. In brief, tissue samples were lysed in RIPA buffer (Bioworld, USA) containing protease and phosphatase inhibitors, and then clarified by centrifugation. Protein concentrations were determined via the BCA assay (Invitrogen, USA). Equal amounts of protein were resolved on 10% SDS–PAGE gels and transferred onto PVDF membranes. Membranes were blocked with 5% nonfat milk for 2 h at room temperature and incubated overnight at 4°C with primary antibodies against MAG (Abcam, USA, ab89780), MBP (Abcam, USA, ab7349), FLAG (CST, USA, 14793S), β‐actin (Bioworld, USA, AP0060), and HDAC2 (Bioworld, USA, BS1162). After washing, membranes were incubated with appropriate HRP‐conjugated secondary antibodies for 2 h at room temperature. Immunoreactive signals were captured using a GelPro imaging system (Tanon Technologies, China), and band intensities were quantified with ImageJ.

For detection of phosphorylated HDAC2 in the SGK1.1‐overexpression experiments, Phos‐tag pre‐cast gels (Wako, 198‐17981) were used to separate phosphorylated and non‐phosphorylated HDAC2 based on their differential electrophoretic mobility. For the age‐course analysis of HDAC2 phosphorylation in 4‐, 5‐, and 6‐month‐old 5 × FAD mice and age‐matched WT controls, Mn^2+^‐Phos‐assay SDS‐PAGE was performed using Phos‐assay Acrylamide (Vazyme, China, PA101). 10% acrylamide resolving gels containing 50 μM Phos‐assay Acrylamide and MnCl_2_ were prepared in a Tris–glycine SDS‐PAGE system according to the manufacturer's instructions. Following electrophoresis, the gels were incubated in transfer buffer containing 10 mM EDTA to chelate Mn^2+^, followed by equilibration in EDTA‐free transfer buffer before protein transfer. Subsequent membrane blocking, antibody incubation, and signal detection were performed as described for conventional Western blotting. The phosphorylated fraction of HDAC2 was calculated as the intensity of the slower‐migrating phosphorylated HDAC2 signal divided by the total HDAC2 signal in the same lane using ImageJ.

### 2.9 Transmission Electron Microscopy (TEM)

Mice were randomly selected from each group, anesthetized and transcardially perfused with phosphate‐buffered saline (PBS). The corpus callosum was rapidly dissected and fixed overnight in 2.5% EM‐grade glutaraldehyde. Subsequent TEM sample processing and image acquisition were carried out by Servicebio (China). Myelination was evaluated by calculating the g‐ratio (axon diameter relative to the diameter of the myelinated fiber) from electron micrographs, and ImageJ was used to measure axon caliber. The g‐ratio and abnormal‐myelin analyses were performed by an investigator blinded to group allocation. For the 5 × FAD experiments, three mice were analyzed per group, with two randomly selected TEM fields of equal size analyzed per animal; 36–56 myelinated axons were analyzed per animal. For g‐ratio analysis, myelinated axons were further stratified according to axon diameter (< 0.8 μm, 0.8–1.2 μm, and > 1.2 μm). In addition to g‐ratio, thinly myelinated axons and axons displaying myelin sheath splitting were quantified by direct visual morphological assessment and counting. For the BCAS experiments, the same blinded sampling procedure was applied, with three mice per group, two TEM fields per mouse, and 31–51 myelinated axons analyzed per animal.

### 2.10 Quantitative Real‐Time PCR (qRT–PCR)

Total RNA was extracted with TRIzol (Vazyme, China) following standard protocols. Residual genomic DNA was eliminated using a DNA wiper mix, and reverse transcription was performed with the PrimeScript RT Reagent Kit (Vazyme, China) according to the manufacturer's instructions to generate cDNA. Quantitative PCR was carried out on a StepOnePlus Real‐Time PCR System (Applied Biosystems, USA) using SYBR Green chemistry (Applied Biosystems, USA) in a 20 μL reaction volume. Isoform‐discriminating primer sets were used to separately quantify canonical *Sgk1* and *Sgk1.1*, using distinct forward primers and a common reverse primer. Accordingly, the canonical *Sgk1* assay does not include *Sgk1.1* [45]. Primer sequences were as follows:

*Sgk1*‐F: CGTCAAAGCCGAGGCTGCTCGAAGC.

*Sgk1*‐R: GGTTTGGCGTGAGGGTTGGAGGAC.

*Sgk1.1*‐F: GAAGGCGGATCGGGATACAGATGCAGTAA.

*Sgk1.1*‐R: GGTTTGGCGTGAGGGTTGGAGGAC.

*Myrf*‐F: CCTGTGTCCGTGGTACTGTG.

*Myrf*‐R: TCACACAGGCGGTAGAAGTG.

*Mag*‐F: CTGCCGCTGTTTTGGATAATGA.

*Mag*‐R: CATCGGGGAAGTCGAAACGG.

*Mbp*‐F: GGCCAGTAAGGATGGAGAGAT.

*Mbp*‐R: CCTCTGAGGCCGTCTGAGA.

*Cnp*‐F: CTCTACTTTGGCTGGTTCCTGAC.

*Cnp*‐R: GCTTCTCCTTGGGTTCATCTCC.

*Actb*‐F: CATTGCTGACAGGATGCAGAAGG.

*Actb*‐R: TGCTGGAAGGTGGACAGTGAGG.

### 2.11 Phosphoproteomics and Bioinformatic Analysis

Quantitative phosphoproteomic profiling was performed on the corpus callosum of 5 × FAD mice following AAV‐mediated *Sgk1.1* overexpression to identify SGK1.1‐responsive phosphorylation changes. All subsequent procedures for sample preparation, LC–MS/MS acquisition, and downstream bioinformatic analyses were performed by Maiwei Metabolism Biotechnology Co. Ltd. (China). Differentially phosphorylated sites were identified using a Fold Change > 1.8 and *p* < 0.01. Gene Ontology (GO) enrichment analysis was subsequently employed to functionally annotate the candidate proteins, which identified HDAC2 as a prioritized effector.

### 2.12 Cell Culture

Primary cortical neurons were obtained from embryonic B6 mouse brains at E15–17 in accordance with an established method [46]. Cells were maintained in Neurobasal medium supplemented with B27 (Invitrogen, USA) and 25 nM glutamine at 37°C in a humidified incubator with 5% CO_2_. Cells were subsequently harvested for downstream experiments. Half of the culture medium was refreshed every 2 days.

Glial cells were isolated from postnatal Day 1 C57BL/6J mice. Both microglia and astrocytes were grown in Dulbecco's modified Eagle's medium (Invitrogen, USA) containing 10% fetal bovine serum (HyClone, Logan, UT, USA) and antibiotics (100 U mL^−1^) under standard culture conditions (37°C, 5% CO_2_). After 10 days of incubation, culture flasks were subjected to orbital shaking (180 rpm, 10 min), allowing microglia to be detached from astrocytes. The resulting cells were then collected for further analysis.

Oligodendrocyte precursor cells were maintained for 8 days in DMEM/F12 (Invitrogen) supplemented with 2% B27, 100 U mL ^−1^ antibiotics, 30 ng mL ^−1^ PDGF (Thermo Fisher Scientific, USA), and 10 ng mL ^−1^ FGF (Thermo Fisher Scientific, USA) for proliferation. To initiate differentiation, cultures were subsequently exposed to a basal DMEM/F12 medium containing 2% B27, 100 U mL ^−1^ antibiotics, 50 ng mL ^−1^ T3 (Thermo Fisher Scientific, USA), and 20 ng mL ^−1^ CNTF (Thermo Fisher Scientific, USA). For viral manipulation during differentiation, on Day 1 cells were transduced with the AAV‐MBP‐3 × FLAG‐Sgk1.1‐EGFP (AAV‐MBP‐EGFP in parallel) virus at a multiplicity of infection (MOI) of 40,000, and the infection medium was replaced 16 h post‐transduction. On Day 2, cells were transduced with lentiviral vectors encoding WT or mutant HDAC2 constructs at a MOI of 20 in the presence of 10 μg mL ^−1^ polybrene, followed by medium replacement 18 h later. Cultures were maintained with half‐medium replacement every 2 days, and all samples were harvested after 7 days for subsequent analyses.

### 2.13 Co‐Immunoprecipitation (Co‐IP)

Total cell lysates from primary oligodendrocyte‐lineage cells transduced with an AAV‐MBP‐3 × FLAG‐Sgk1.1‐EGFP virus were prepared using RIPA buffer containing protease inhibitors. Lysates were clarified by centrifugation and then pre‐cleared with Protein A/G PLUS agarose beads (Millipore, USA) to minimize non‐specific binding. The cleared lysates were then incubated with anti‐FLAG beads (AlpaLifeBio, KTSM1361) for 2 h at 4°C with rotation. In parallel, the same lysate was incubated with beads alone as a beads‐only negative control. After incubation, the beads were washed 3 times with dilution buffer to remove unbound proteins. For detection, the beads were resuspended in loading buffer, heated to 95°C for 10 min, and subjected to Western blotting. To detect HDAC2, the membrane was incubated with primary anti‐HDAC2 antibody (Bioworld, BS1162) and anti‐FLAG (CST, 14793S) antibody, respectively, followed by appropriate HRP‐conjugated secondary antibodies. The bands were visualized using the GelPro imaging system (Tanon Technologies). Three independent Co‐IP experiments were performed. HDAC2 recovered in the FLAG‐immunoprecipitated fraction was quantified relative to the corresponding FLAG signal, and the HDAC2/FLAG ratio was used for quantitative analysis.

### 2.14 Statistics

Statistical analyses were conducted using SPSS version 22.0 (SPSS Inc., USA) and GraphPad Prism 8 (GraphPad Software, USA). Data are presented as mean ± standard error of the mean (SEM). Data distribution was assessed using the Shapiro–Wilk test. For normally distributed data, comparisons between two groups were performed using a two‐tailed Student's *t*‐test, and comparisons across multiple groups were analyzed using one‐way ANOVA followed by Tukey's post hoc test. For non‐normally distributed multiple‐group data, the Kruskal–Wallis test followed by Dunn's post hoc test was used. A *p*‐value of less than 0.05 was considered statistically significant. The specific statistical tests applied for each analysis are described in the figure legends.

## 3 Results

### 3.1 Oligodendroglial *Sgk1.1* mRNA Elevation Precedes Demyelination in 5 × FAD Mice

**FIGURE 1:**
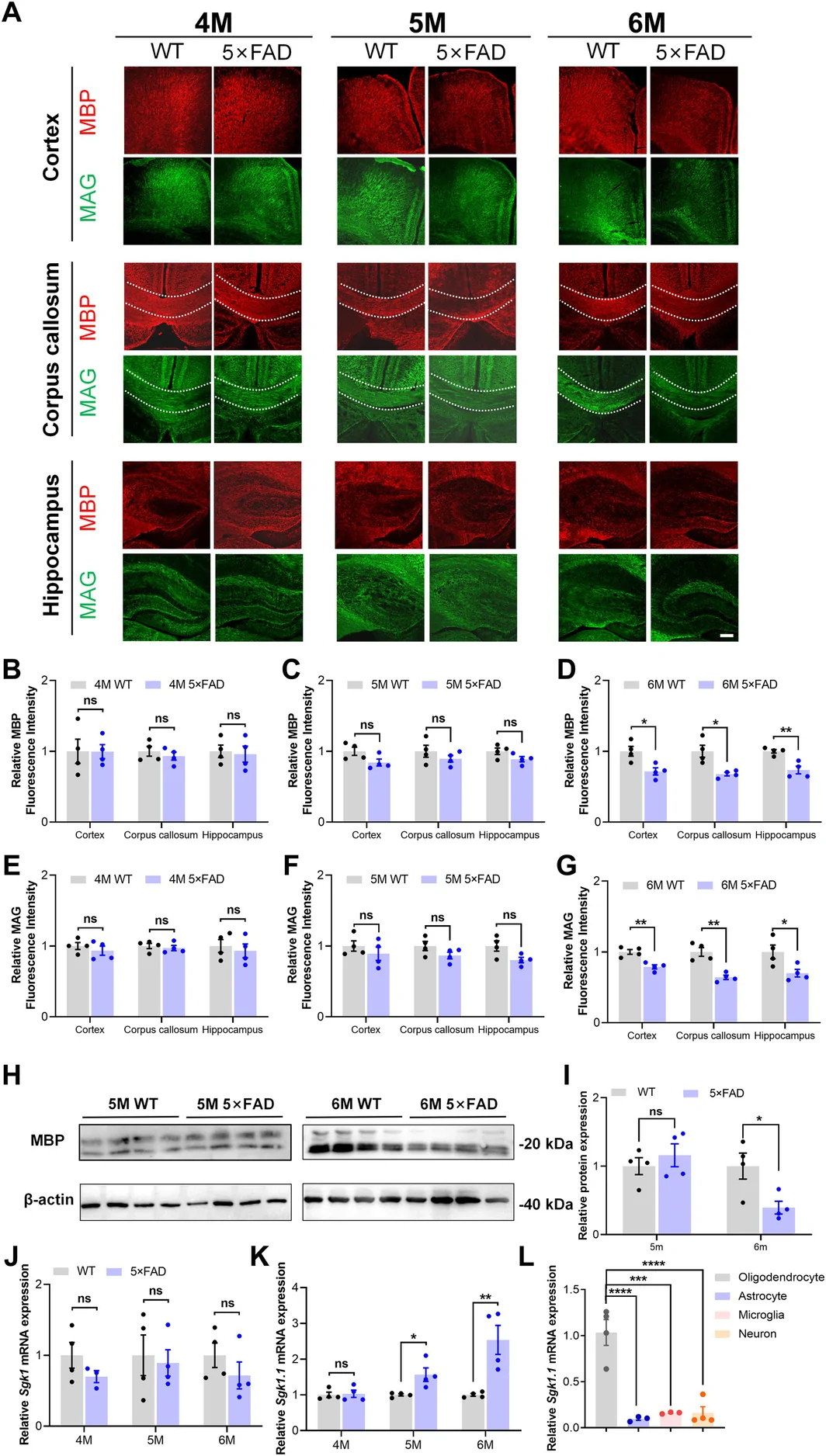
Early *Sgk1.1* transcript elevation in the corpus callosum precedes myelin impairment in 5 × FAD mice. (A) Representative immunofluorescence images of MBP and MAG in the cortex, corpus callosum, and hippocampus of 4‐, 5‐, and 6‐month‐old 5 × FAD mice and age‐matched littermate WT controls. Scale bar, 200 μm. (B–G) Quantification of MBP and MAG immunofluorescence intensity in the cortex, corpus callosum, and hippocampus of 4‐, 5‐, and 6‐month‐old 5 × FAD mice and WT mice. Fluorescence intensities were normalized to the mean value of the WT group. *n* = 4 mice per group. (H–I) Representative immunoblot bands and quantification of MBP protein levels in the corpus callosum of 5‐ and 6‐month‐old 5 × FAD mice and WT mice. Band intensities were normalized to β‐actin and then normalized to the mean value of the corresponding control group. *n* = 4 mice per group. (J) Quantitative PCR analysis of *Sgk1* mRNA expression in the corpus callosum of 4‐, 5‐, and 6‐month‐old 5 × FAD mice and WT mice. Expression levels were normalized to *Actb* and presented relative to the corresponding age‐matched WT group. *n* = 3–4 mice per group. (K) Quantitative PCR analysis of *Sgk1.1* mRNA expression in the corpus callosum of 4‐, 5‐, and 6‐month‐old 5 × FAD mice and littermate WT controls. Expression levels were normalized to *Actb* and presented relative to the corresponding age‐matched WT group. *n* = 4 mice per group. (L) Quantitative PCR analysis of *Sgk1.1* mRNA expression in primary cultured neural cells (oligodendrocytes, astrocytes, microglia, and neurons). Expression levels were normalized to *Actb* and presented relative to the oligodendrocyte group. *n* = 3–4 per group. Data are presented as mean ± SEM. *p*‐values were determined by two‐tailed Student's *t*‐test in B–G and I–K, and by one‐way ANOVA with Tukey's post hoc analysis in L. ns, not significant; *p < 0.05, **p < 0.01, ***p < 0.001, ****p < 0.0001.

To define the temporal sequence between SGK1 isoform expression and WM pathology, we examined myelin integrity and *Sgk1* isoform expression from early stages to the onset of cognitive impairment in 5 × FAD mice. Immunofluorescence analyses showed that the expression of myelin‐associated proteins (MBP and MAG) in the cortex, corpus callosum, and hippocampus of 5 × FAD mice was largely preserved at 4 and 5 months of age compared with age‐matched WT controls. In contrast, by 6 months of age, both MBP and MAG signals were robustly reduced across these regions, indicating the onset of demyelination (Figure 1A–G). Western blotting analyses further confirmed a significant reduction in MBP protein expression at 6 months, but not at earlier stages (Figure 1H,I).

We next asked whether either SGK1 isoform showed an early disease‐associated change preceding overt myelin impairment. Using isoform‐discriminating quantitative PCR primers that distinguish canonical *Sgk1* from the brain‐enriched splice isoform *Sgk1.1* [45], we found that canonical *Sgk1* mRNA levels remained relatively stable across 4–6 months of age in 5 × FAD mice compared with WT controls. In contrast, *Sgk1.1* mRNA levels were significantly increased at 5 months and remained elevated at 6 months, preceding myelin disruption (Figure 1J,K). This differential temporal pattern prompted us to prioritize *Sgk1.1* for subsequent cell‐type‐resolved and functional analyses.

To obtain an initial indication of the cell types potentially associated with *Sgk1.1* expression, we isolated and cultured primary brain cell types from mice, including oligodendrocyte lineage cells, neurons, astrocytes, and microglia, and then assessed *Sgk1.1* mRNA levels by quantitative PCR. The results revealed that S*gk1.1* mRNA levels were higher in cultured oligodendrocyte lineage cells than in the other neural cell types examined (Figure 1L).

**FIGURE 2:**
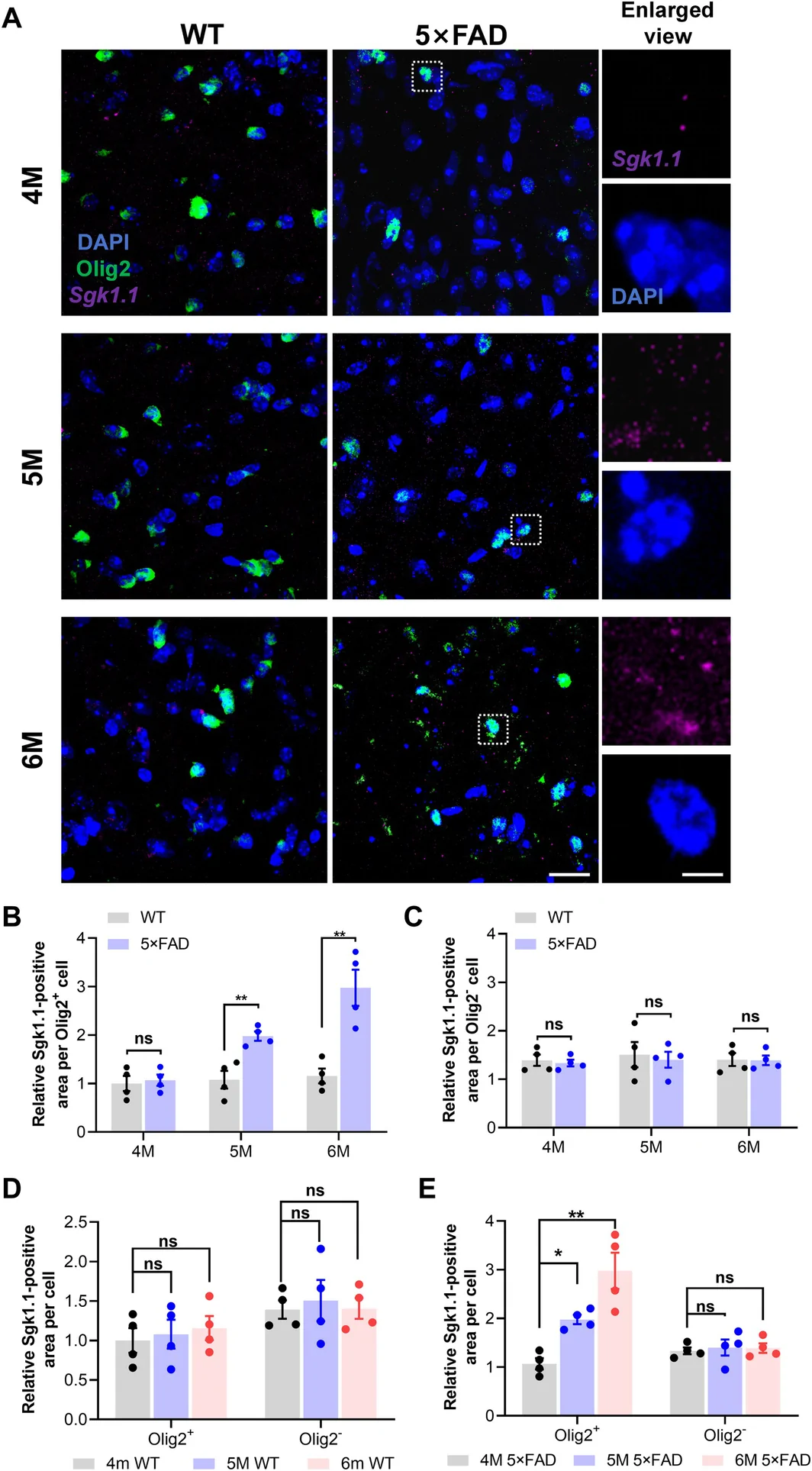
*Sgk1.1* mRNA is elevated in cortical Olig2^+^ cells of 5 × FAD mice at 5 and 6 months of age. (A) Representative images of RNAscope in situ hybridization for *Sgk1.1* mRNA combined with Olig2 immunofluorescence and DAPI nuclear counterstaining in the cortex of 4‐, 5‐, and 6‐month‐old 5 × FAD mice and age‐matched littermate WT controls. Enlarged views show selected Olig2^+^ cells with detectable *Sgk1.1* signal and corresponding DAPI images. Scale bars, 17 μm (overview) and 1.7 μm (enlarged view). (B, C) Quantification of the relative *Sgk1.1*‐positive area per Olig2^+^ cell (B) or Olig2^−^ cell (C) in age‐matched WT and 5 × FAD mice. All values were normalized to the mean value of the Olig2^+^ cells from 4‐month‐old WT mice. *n* = 4 mice per group. (D, E) Quantification of the relative *Sgk1.1*‐positive area per Olig2^+^ or Olig2^−^ cell across 4, 5, and 6 months of age in WT mice (D) and 5 × FAD mice (E). All values were normalized to the mean value of the Olig2^+^ cells from 4‐month‐old WT mice. *n* = 4 mice per group. Data are presented as mean ± SEM. *p*‐values were determined by two‐tailed Student's *t*‐test for the WT versus 5 × FAD comparison at each age in B, C. Comparisons across ages in D, E were performed separately within the Olig2^+^ and Olig2^−^ compartments using one‐way ANOVA with Tukey's post hoc analysis. ns, not significant; *p < 0.05, **p < 0.01.

To determine whether the disease‐associated increase in *Sgk1.1* mRNA was associated with the oligodendrocyte lineage in vivo, we next performed *Sgk1.1*‐specific RNAscope combined with Olig2 immunofluorescence. In the cortex, the Olig2^+^‐associated *Sgk1.1* mRNA signal was comparable between 5 × FAD mice and age‐matched WT mice at 4 months, but was significantly increased in 5 × FAD mice at 5 and 6 months (Figure 2A,B). In contrast, no significant disease‐associated increase was detected in the Olig2^−^ compartment across the same ages (Figure 2C). Analysis of the temporal changes within each cellular compartment further showed that the age‐associated increase in *Sgk1.1* mRNA in 5 × FAD mice occurred predominantly within the Olig2^+^ compartment (Figure 2D,E). A similar pattern was observed in the corpus callosum, where *Sgk1.1* mRNA was likewise increased in Olig2^+^ cells at 5 and 6 months without a significant disease‐associated increase in the Olig2^−^ compartment (Figure S1).

Together, these findings indicate that an early increase in *Sgk1.1* mRNA precedes overt demyelination in 5 × FAD mice and is predominantly detected in oligodendrocyte‐lineage cells.

### 3.2 Oligodendrocyte Lineage–Targeted *Sgk1.1* Knockdown Ameliorates Cognitive Deficits in 5 × FAD Mice

**FIGURE 3:**
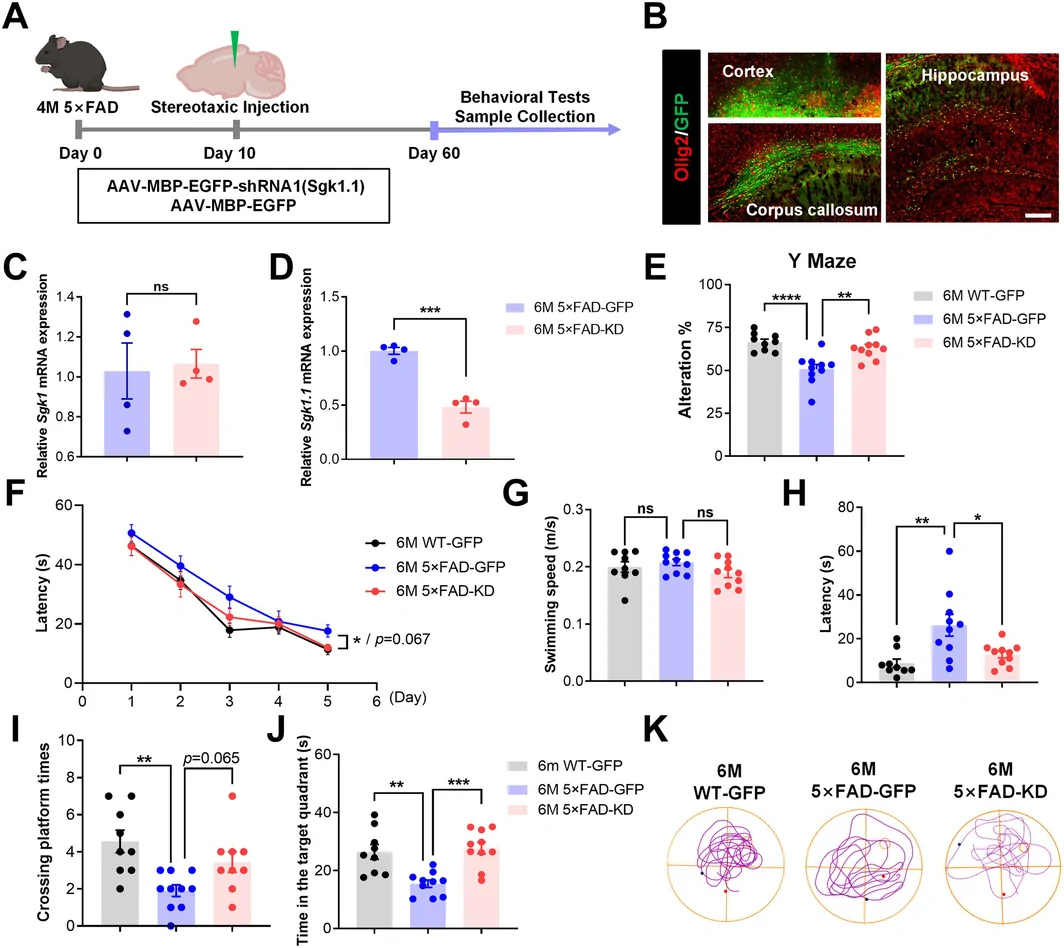
Oligodendrocyte‐targeted knockdown of *Sgk1.1* ameliorates cognitive impairment in 5 × FAD mice. (A) Experimental timeline and schematic of stereotaxic AAV injection in 5 × FAD mice, followed by behavioral tests and tissue collection. (B) Representative immunofluorescence images showing viral transduction (GFP) and co‐localization with the oligodendrocyte lineage marker Olig2 in the cortex, corpus callosum, and hippocampus. Scale bar, 200 μm. (C) Quantitative PCR analysis of *Sgk1* mRNA expression in the corpus callosum of 6‐month‐old 5 × FAD mice injected with the *Sgk1.1*‐knockdown virus (5 × FAD‐KD) or control virus (5 × FAD‐GFP). Expression was normalized to *Actb* and presented relative to the 5 × FAD‐GFP group. *n* = 4 mice per group. (D) Quantitative PCR analysis of *Sgk1.1* mRNA expression in the corpus callosum of 6‐month‐old 5 × FAD‐KD and 5 × FAD‐GFP mice. Expression was normalized to *Actb* and presented relative to the 5 × FAD‐GFP group. *n* = 4 mice per group. (E) Y‐maze spontaneous alternation (%) of 6‐month‐old WT mice injected with control virus (WT‐GFP), 5 × FAD‐GFP mice, and 5 × FAD‐KD mice. *n* = 10 mice per group. (F–K) Morris water maze performance of 6‐month‐old WT‐GFP, 5 × FAD‐GFP, and 5 × FAD‐KD mice, including escape latency across training days (F), swimming speed (G), latency to first entry into the target quadrant during the probe trial (H), platform crossing times (I), time spent in the target quadrant during the probe trial (J), and representative swim paths in the probe trial (K). *n* = 10 mice per group. Data are presented as mean ± SEM. *p*‐values were determined by two‐tailed Student's *t*‐test in C and D, and by one‐way ANOVA with Tukey's post hoc analysis in E, the Day 5 escape latency in F, and G–J. ns, not significant; *p < 0.05, **p < 0.01.

To test whether the early increase of oligodendrocyte‐associated *Sgk1.1* mRNA contributed to cognitive impairment, we selectively knocked down *Sgk1.1* in oligodendrocyte lineage cells using an MBP promoter–driven AAV strategy, consistent with a previously validated oligodendrocyte‐lineage–enriched gene delivery tool [48]. AAV‐MBP‐EGFP‐shRNA1(Sgk1.1) virus or the control virus was stereotaxically injected into the hippocampus, cortex, and corpus callosum of 4‐month‐old 5 × FAD mice to ensure effective *Sgk1.1* knockdown during the window of its aberrant upregulation (Figure 3A). Viral targeting specificity was confirmed by immunofluorescence staining, which showed robust transduction within oligodendrocyte lineage cells (Figure 3B). Quantitative PCR analyses further demonstrated a significant reduction in *Sgk1.1* mRNA, while canonical *Sgk1* mRNA expression was largely preserved, indicating that the intervention preferentially counteracted the disease‐associated *Sgk1.1* mRNA elevation rather than broadly suppressing canonical *Sgk1* expression (Figure 3C,D).

To further validate the effect of *Sgk1.1* knockdown at the endogenous transcript level within oligodendrocyte‐lineage cells, we performed *Sgk1.1*‐specific RNAscope combined with Olig2 immunofluorescence staining in 6‐month‐old 5 × FAD‐GFP and 5 × FAD‐KD mice. *Sgk1.1* mRNA signal associated with Olig2^+^ cells was significantly reduced after *Sgk1.1* knockdown in both the cortex and corpus callosum, whereas no significant change was detected in the Olig2^−^ compartment (Figure S2).

We next assessed cognitive performance at 6 months of age, a time point when 5 × FAD mice display measurable cognitive deficits [49]. Working memory was first evaluated using the Y‐maze test. Control‐treated 5 × FAD mice exhibited a significant reduction in spontaneous alternation rates compared with WT littermates. Conversely, *Sgk1.1* knockdown markedly increased the alternation performance of 5 × FAD mice (Figure 3E). Spatial learning and memory were further evaluated using the Morris water maze. During the acquisition phase, control‐treated 5 × FAD mice exhibited impaired learning acquisition compared with WT mice, as reflected by a slower reduction in escape latency across training sessions. In contrast, 5 × FAD mice with *Sgk1.1* knockdown showed a trend toward improved spatial navigation learning (Figure 3F). In the subsequent probe trial, control‐treated 5 × FAD mice displayed impaired spatial memory relative to WT mice. By comparison, *Sgk1.1* knockdown improved spatial memory performance of 5 × FAD mice (Figure 3G–K), supporting a rescue of spatial memory deficits. Notably, swimming speeds were comparable between groups, excluding motor impairment as a confounding factor.

### 3.3 *Sgk1.1* Knockdown Preserves Myelin Integrity and Rescues Oligodendrocyte Maturation Deficits in 5 × FAD Mice

**FIGURE 4:**
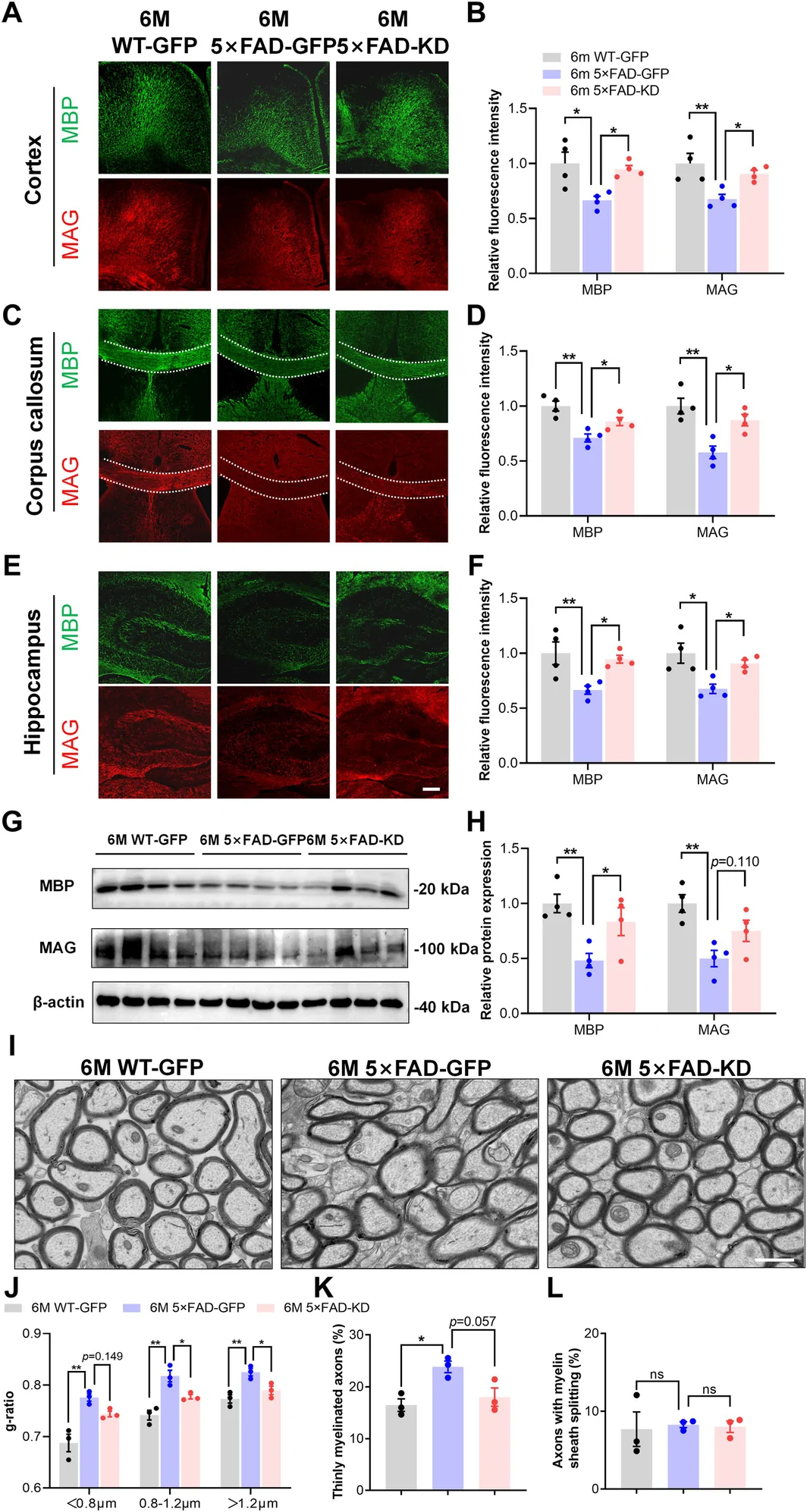
Oligodendrocyte‐targeted knockdown of *Sgk1.1* alleviates myelin impairment in 5 × FAD mice. (A) Representative immunofluorescence images of MBP and MAG in the cortex of 6‐month‐old WT‐GFP, 5 × FAD‐GFP, and 5 × FAD‐KD mice. Scale bar, 200 μm. (B) Quantification of MBP and MAG immunofluorescence intensity in the cortex of mice. Fluorescence intensities were normalized to the mean value of the WT‐GFP group. *n* = 4 mice per group. (C) Representative immunofluorescence images of MBP and MAG in the corpus callosum across the indicated experimental groups. Scale bar, 200 μm. (D) Quantification of MBP and MAG immunofluorescence intensity in the corpus callosum of mice. Fluorescence intensities were normalized to the mean value of the WT‐GFP group. *n* = 4 mice per group. (E) Representative immunofluorescence images of MBP and MAG in the hippocampus across the indicated experimental groups. Scale bar, 200 μm. (F) Quantification of MBP and MAG immunofluorescence intensity in the hippocampus of mice. Fluorescence intensities were normalized to the mean value of the WT‐GFP group. *n* = 4 mice per group. (G) Representative immunoblot bands of MBP and MAG in corpus callosum lysates from 6‐month‐old WT‐GFP, 5 × FAD‐GFP, and 5 × FAD‐KD mice. (H) Quantification of MBP and MAG protein levels in the corpus callosum of mice. Band intensities were normalized to β‐actin and then normalized to the mean value of the WT‐GFP group. *n* = 4 mice per group. (I) Representative TEM images of corpus callosum from 6‐month‐old WT‐GFP, 5 × FAD‐GFP, and 5 × FAD‐KD mice. Scale bar, 1 μm. (J) Quantification of g‐ratio in the indicated experimental groups, grouped by axon diameter. *n* = 3 mice per group. (K) Quantification of the percentage of thinly myelinated axons in the indicated experimental groups. *n* = 3 mice per group. (L) Quantification of the percentage of myelinated axons displaying myelin sheath splitting in the indicated experimental groups. *n* = 3 mice per group. Data are presented as mean ± SEM. *p*‐values were determined by one‐way ANOVA with Tukey's post hoc analysis in B, D, F, H, and J–L. ns, not significant; *p < 0.05, **p < 0.01, ***p < 0.001.

Given the behavioral benefits of *Sgk1.1* knockdown, we next evaluated white matter pathology in treated 5 × FAD mice at 6 months of age. Immunofluorescence staining revealed that control‐treated 5 × FAD mice displayed marked reductions of MBP and MAG expression in the cortex, corpus callosum, and hippocampus. Notably, *Sgk1.1* knockdown significantly restored MBP and MAG signal intensity across these regions (Figure 4A–F). Consistent with these findings, Western blotting analyses confirmed a partial recovery of MBP and MAG protein abundance in *Sgk1.1* knockdown mice relative to control‐treated counterparts (Figure 4G,H).

To assess whether these changes extended to myelin ultrastructure, we performed transmission electron microscopy on the corpus callosum. Compared with WT mice, control‐treated 5 × FAD mice exhibited thinner myelin sheaths, as evidenced by increased g‐ratios. *Sgk1.1* knockdown shifted g‐ratio distributions toward WT levels, indicative of improved myelin thickness and structural integrity (Figure 4I,J). Additional morphological analysis showed that the percentage of thinly myelinated axons was significantly increased in control‐treated 5 × FAD mice compared with WT mice and showed a trend toward reduction following *Sgk1.1* knockdown (Figure 4K). In contrast, the percentage of axons displaying myelin sheath splitting did not differ significantly among the three groups (Figure 4L).

**FIGURE 5:**
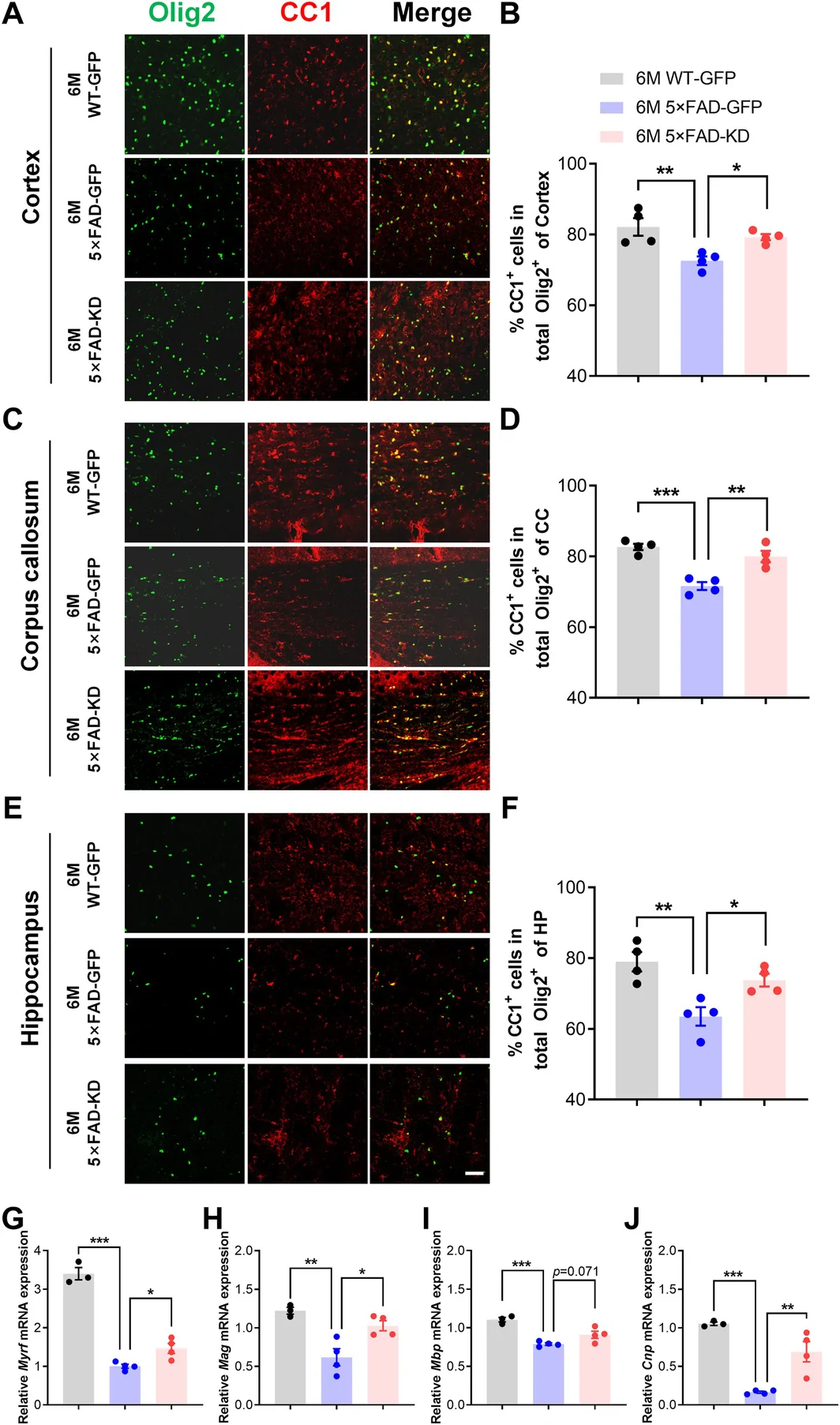
Oligodendrocyte‐targeted knockdown of *Sgk1.1* rescues impaired oligodendrocyte maturation in 6‐month‐old 5 × FAD mice. (A) Representative immunofluorescence images of Olig2 and CC1 in the cortex of 6‐month‐old WT‐GFP, 5 × FAD‐GFP, and 5 × FAD‐KD mice. Scale bar, 50 μm. (B) Quantification of oligodendrocyte maturation in the cortex, expressed as the percentage of CC1^+^ cells among Olig2^+^ cells (CC1^+^/Olig2^+^). *n* = 4 mice per group. (C) Representative immunofluorescence images of Olig2 and CC1 in the corpus callosum of 6‐month‐old WT‐GFP, 5 × FAD‐GFP, and 5 × FAD‐KD mice. Scale bar, 50 μm. (D) Quantification of oligodendrocyte maturation in the corpus callosum, expressed as the percentage of CC1^+^ cells among Olig2^+^ cells (CC1^+^/Olig2^+^). *n* = 4 mice per group. (E) Representative immunofluorescence images of Olig2 and CC1 in the hippocampus of 6‐month‐old WT‐GFP, 5 × FAD‐GFP, and 5 × FAD‐KD mice. Scale bar, 50 μm. (F) Quantification of oligodendrocyte maturation in the hippocampus, expressed as the percentage of CC1^+^ cells among Olig2^+^ cells (CC1^+^/Olig2^+^). *n* = 4 mice per group. (G–J) Quantitative PCR analysis of *Myrf* (G), *Mag* (H), *Mbp* (I), and *Cnp* (J) mRNA expression in the corpus callosum of 6‐month‐old WT‐GFP, 5 × FAD‐GFP, and 5 × FAD‐KD mice. Gene expression was normalized to *Actb* and then normalized to the mean value of the WT‐GFP group. *n* = 3–4 mice per group. Data are presented as mean ± SEM. *p*‐values were determined by one‐way ANOVA with Tukey's post hoc analysis in B, D, F, and G–J. ns, not significant; *p < 0.05, **p < 0.01, ***p < 0.001.

As proper maturation of oligodendrocyte lineage cells is critical for myelin formation and maintenance [20, 50], we next assessed oligodendrocyte differentiation state. Co‐immunostaining for the oligodendrocyte lineage marker Olig2 and the mature oligodendrocyte marker CC1 revealed a diminished proportion of mature oligodendrocytes (CC1^+^/Olig2^+^) in control‐treated 5 × FAD mice across multiple brain regions, suggesting impaired oligodendrocyte maturation in AD mice. *Sgk1.1* knockdown significantly increased the fraction of mature oligodendrocytes (Figure 5A–F), reflecting an alleviation of the maturation deficit. Consistently, total Olig2^+^ cell density was unchanged, whereas PDGFRα^+^/Olig2^+^ OPC density was increased in 5 × FAD mice and partially reduced by *Sgk1.1* knockdown (Figure S3), further supporting impaired oligodendrocyte maturation.

In addition, qPCR analyses showed that, relative to WT, 5 × FAD mice exhibited markedly reduced transcript levels of oligodendrocyte differentiation–associated genes in the corpus callosum, including *Myrf*, *Mag*, *Mbp*, and *Cnp*. Notably, *Sgk1.1* knockdown restored the expression of these genes, consistent with improved oligodendrocyte maturation (Figure 5G–J).

### 3.4 *Sgk1.1* Overexpression Impairs Myelin‐Associated Features In Vitro

To further determine whether *Sgk1.1* overexpression is sufficient to impair oligodendrocyte differentiation, we performed in vitro gain‐of‐function experiments. Primary OPCs were isolated from neonatal mice and expanded under proliferative conditions for 8 days. At the onset of differentiation, cells were transduced with an MBP promoter–driven AAV expressing *Sgk1.1* or the control vector, followed by differentiation induction for 8 days (Figure S4A).

Viral transduction was confirmed by GFP immunofluorescence, and quantitative PCR analysis showed increased *Sgk1.1* mRNA without affecting canonical *Sgk1* mRNA (Figure S4B–D). Subsequent Western blotting analyses showed that *Sgk1.1* overexpression markedly reduced the protein levels of myelin‐associated markers MAG and MBP (Figure S4E,F). In parallel, quantitative PCR analyses demonstrated that *Sgk1.1* overexpression significantly suppressed the expression of *Myrf, Mag, Mbp*, and *Cnp*, compared with control group (Figure S4G–J).

Together with the in vivo knockdown data, these findings support that elevated *Sgk1.1* suppresses oligodendrocyte maturation‐associated molecular features in vitro.

### 3.5 HDAC2 S422/S424 Phosphorylation Mediates SGK1.1‐Associated Suppression of Oligodendrocyte Maturation

**FIGURE 6:**
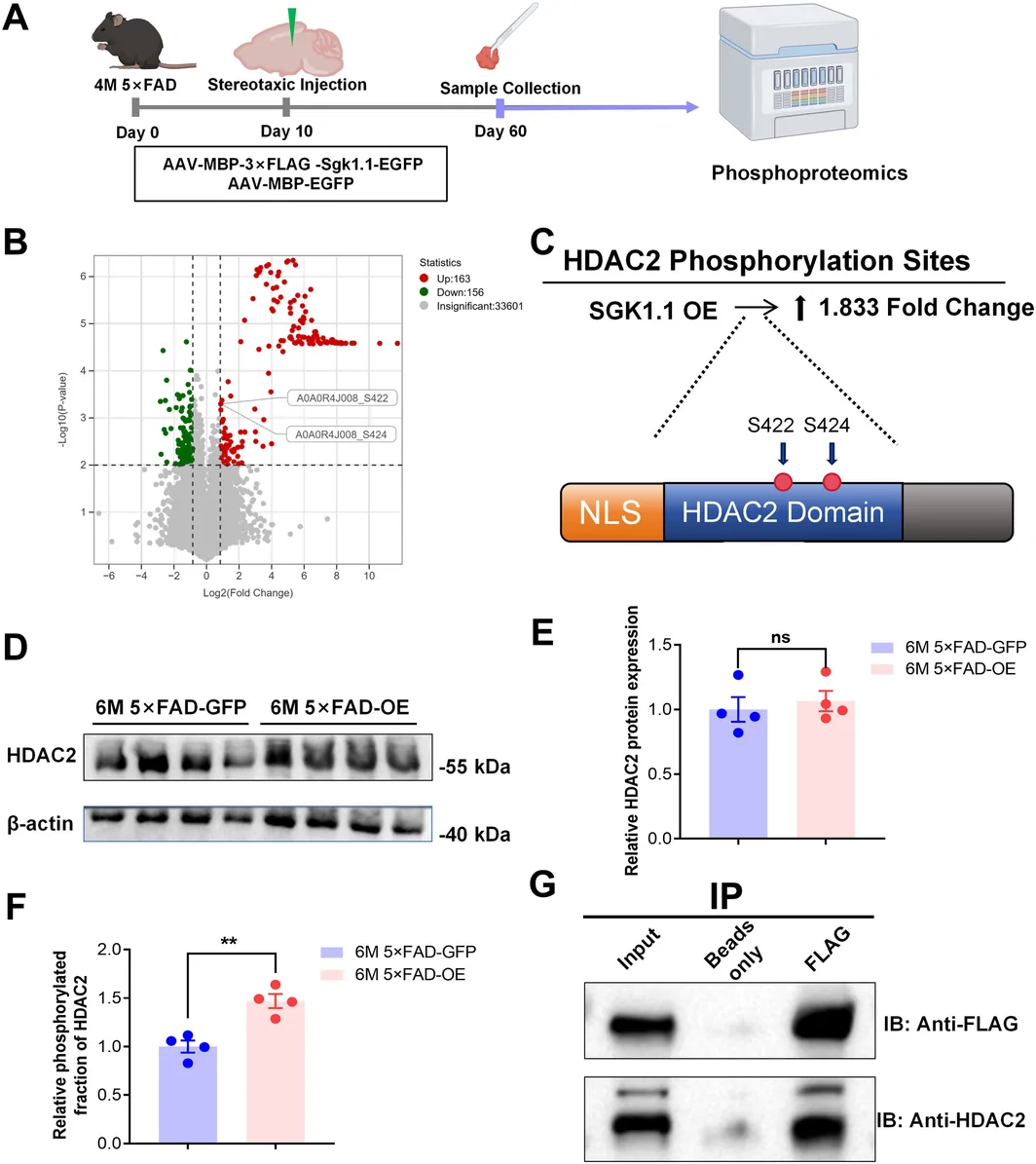
*Sgk1.1* overexpression promotes HDAC2 S422/S424 phosphorylation. (A) Schematic illustration of the experimental workflow for phosphoproteomics. (B) Volcano plot of the phosphoproteomics dataset comparing the OE and control groups. The highlighted dots indicate two HDAC2 phosphosites with increased phosphorylation upon *Sgk1.1* overexpression. *n* = 3–4 mice per group. (C) Schematic diagram of HDAC2 showing the phosphorylation sites upregulated in the phosphoproteomics analysis following *Sgk1.1* overexpression. *n* = 3–4 mice per group. (D–F) Representative immunoblot bands of HDAC2 resolved by Phos‐tag SDS‐PAGE (D) and quantification of total HDAC2 protein levels (E) and the phosphorylated fraction of HDAC2 (F) in corpus callosum lysates from 5 × FAD‐GFP mice and 5 × FAD‐OE mice. Band intensities for total HDAC2 were normalized to β‐actin, whereas the phosphorylated fraction was calculated as the intensity of the slower‐migrating phosphorylated HDAC2 signal divided by the total HDAC2 signal in the same lane. Values were then normalized to the mean value of the 5 × FAD‐GFP group. *n* = 4 mice per group. (G) Co‐IP analysis of the association between SGK1.1 and HDAC2 in primary oligodendrocytes transduced with SGK1.1‐FLAG. Whole‐cell lysates were loaded as Input. Immunoprecipitation was performed with anti‐FLAG to pull down SGK1.1‐FLAG, and a beads‐only negative control was included. Input and precipitates were immunoblotted with anti‐FLAG and anti‐HDAC2 antibodies. Data are presented as mean ± SEM. *p*‐values were determined by two‐tailed Student's *t*‐test in E, F. ns, not significant; *p < 0.05, **p < 0.01, ***p < 0.001, ****p < 0.0001.

Given that SGK1.1 is a serine/threonine kinase, we next sought to elucidate phosphorylation‐dependent downstream events that may mediate its effects on oligodendrocyte maturation. To this end, we stereotaxically injected an AAV‐MBP‐3 × FLAG‐Sgk1.1‐EGFP or control virus into the corpus callosum of 4‐month‐old 5 × FAD mice to achieve oligodendrocyte‐targeted *Sgk1.1* overexpression. Corpus callosum tissues were harvested at 6 months of age for quantitative phosphoproteomic profiling to identify SGK1.1‐responsive phosphorylation changes (Figure 6A).

Comparative phosphoproteomic analysis identified 163 phosphosites that were significantly upregulated upon *Sgk1.1* overexpression (fold change > 1.8, *p* < 0.01). Gene Ontology (GO) enrichment analysis highlighted HDAC2 as the sole protein associated with ‘positive regulation of oligodendrocyte differentiation’ (GO:0048714) among the candidates exhibiting increased phosphorylation. Notably, phosphorylation at HDAC2 Ser422 and Ser424 was elevated in the *Sgk1.1*‐overexpressing condition (Figure 6B,C and Table S1), positioning HDAC2 phosphorylation as a potential downstream effector of SGK1.1 signaling in oligodendrocytes.

To further investigate the relationship between SGK1.1 and HDAC2, we first assessed whether SGK1.1 influences HDAC2 protein abundance. Phos‐tag analysis of corpus callosum tissues from 5 × FAD mice showed that *Sgk1.1* overexpression increased the phosphorylated fraction of HDAC2 without altering total HDAC2 protein levels (Figure 6D–F), supporting an association between *Sgk1.1* overexpression and increased HDAC2 phosphorylation.

We next examined the temporal profile of HDAC2 phosphorylation during disease progression. The phosphorylated fraction of HDAC2 was unchanged at 4 months, showed an upward trend at 5 months, and was significantly increased at 6 months in 5 × FAD mice, whereas total HDAC2 levels remained unchanged across these ages (Figure S5). This progressive pattern broadly paralleled the age‐associated increase in *Sgk1.1* mRNA observed during the same disease window.

At the protein‐interaction level, co‐immunoprecipitation revealed an association between SGK1.1 and HDAC2 in oligodendrocyte lineage cells, which was consistently observed across independent experiments (Figures 6G and S6). Together, these findings support HDAC2 phosphorylation as a downstream event associated with SGK1.1 signaling in oligodendrocytes.

**FIGURE 7:**
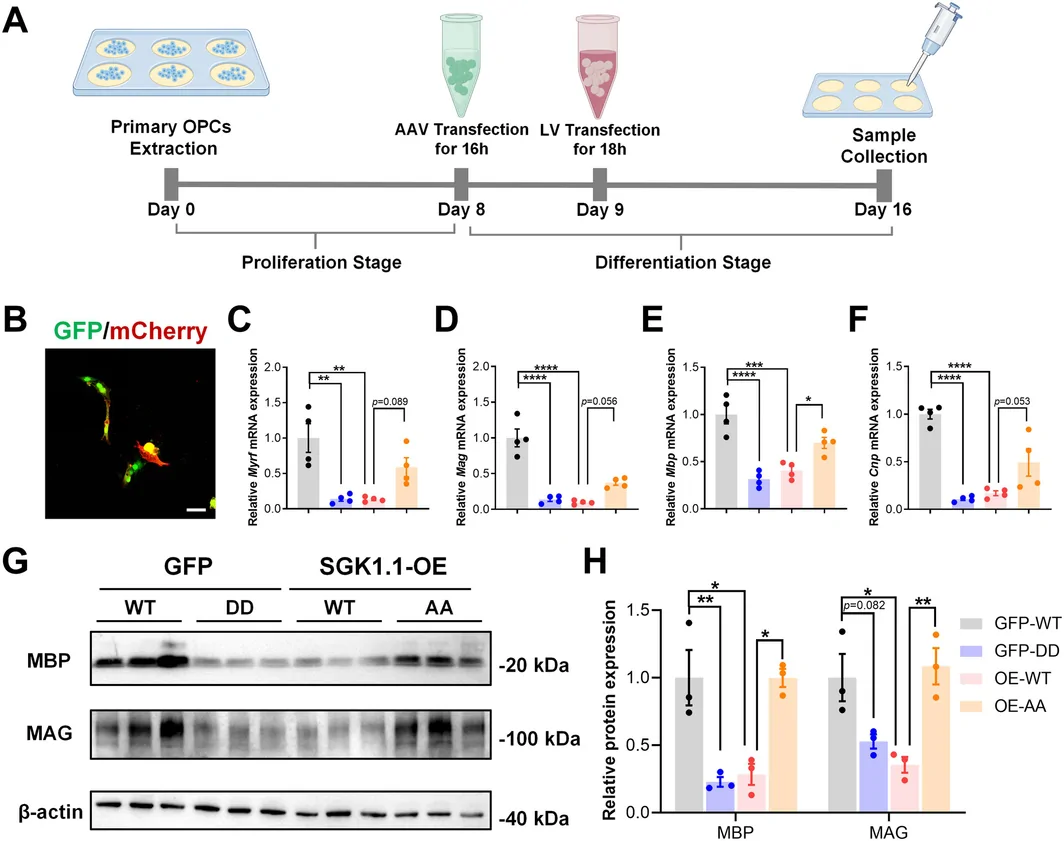
HDAC2 S422/S424 phosphorylation mediates *Sgk1.1*‐driven suppression of oligodendrocyte differentiation in primary OPCs. (A) Experimental workflow for primary OPC culture, viral manipulation, and sample collection. (B) Representative immunofluorescence images showing transduction in primary oligodendrocyte‐lineage cells. GFP indicates AAV‐mediated transduction (control GFP or SGK1.1‐OE), and mCherry indicates lentiviral delivery of HDAC2 constructs. Scale bar, 25 μm. (C–F) Quantitative PCR analysis of *Myrf* (C), *Mag* (D), *Mbp* (E), and *Cnp* (F) across groups. Gene expression was normalized to *Actb* and then normalized to the mean value of the GFP‐WT group. *n* = 4 per group. WT denotes wild‐type HDAC2; DD denotes the phosphomimetic S422D/S424D mutant; and AA denotes the non‐phosphorylatable S422A/S424A mutant. GFP and OE indicate control AAV and *Sgk1.1*‐overexpression AAV, respectively. (G) Representative immunoblots of myelin proteins MBP and MAG in primary oligodendrocyte‐lineage cells across the indicated conditions. β‐actin serves as a loading control. (H) Quantification of MBP and MAG protein levels normalized to β‐actin and further normalized to the mean value of the GFP‐WT group. *n* = 3 per group. Data are presented as mean ± SEM. *p*‐values were determined by one‐way ANOVA with Tukey's post hoc analysis in C–F and H. ns, not significant; *p < 0.05, **p < 0.01, ***p < 0.001, ****p < 0.0001.

To test whether HDAC2 phosphorylation at Ser422 and Ser424 mediates SGK1.1‐driven suppression of oligodendrocyte maturation, phospho‐deficient (S422A/S424A), phospho‐mimetic (S422D/S424D), and wild‐type (WT) HDAC2 constructs were generated and delivered via lentiviral vectors. Primary oligodendrocyte lineage cells were transduced with control or *Sgk1.1*‐overexpression AAV together with the indicated HDAC2 variants (Figure 7A). After validating transduction efficiency through immunofluorescence staining (Figure 7B), the levels of key myelin proteins and the expression of differentiation‐associated genes were quantified. These analyses revealed that overexpression of the phospho‐mimetic S422D/S424D mutant alone was sufficient to phenocopy SGK1.1‐induced deficits, markedly reducing myelin protein levels and suppressing differentiation‐associated genes. Conversely, the phospho‐deficient S422A/S424A mutant antagonized the suppressive effects of SGK1.1, partially restoring the expression of both myelin proteins and differentiation‐associated genes (Figure 7C–H). Together, these data support a functional role for HDAC2 S422/S424 phosphorylation in SGK1.1‐associated suppression of oligodendrocyte maturation.

### 3.6 Oligodendroglial *Sgk1.1* Overexpression Aggravates White Matter Injury and Cognitive Deficits in the Chronic Ischemia Model

To examine whether elevated oligodendroglial *Sgk1.1* also exerts detrimental effects in an independent white matter injury context, we used the bilateral common carotid artery stenosis (BCAS) model, a standard paradigm for white matter injury and related cognitive impairment (Figure S7A). Interestingly, in contrast to the 5 × FAD model, endogenous *Sgk1.1* mRNA expression in the corpus callosum was significantly downregulated two months post‐BCAS surgery, whereas canonical *Sgk1* mRNA levels remained largely unchanged (Figure S7B,C). This distinct expression profile led us to test the consequence of elevating oligodendroglial *Sgk1.1* in BCAS mice.

To this end, the AAV‐MBP–SGK1.1‐EGFP virus was stereotaxically delivered into the corpus callosum of 2‐month‐old C57BL/6J mice. Following viral expression, mice underwent BCAS or sham surgery, with behavioral and pathological assessments conducted 2 months later (Figure S7D). Immunofluorescence staining confirmed oligodendrocyte‐lineage targeting, while quantitative PCR showed increased *Sgk1.1* mRNA without altering canonical *Sgk1* mRNA (Figure S7E–H). In the open‐field test, total travel distance and time spent in each zone were comparable across all groups, indicating no detectable effects of BCAS surgery or *Sgk1.1* overexpression on locomotor activity or anxiety‐like behavior (Figure S7I). In the Y‐maze, BCAS mice exhibited reduced spontaneous alternation relative to sham controls, and this deficit was further exacerbated by *Sgk1.1* overexpression, indicating a significant worsening of working memory (Figure S7J). Similarly, while spatial acquisition in the Morris water maze remained similar across groups, *Sgk1.1* overexpression aggravated spatial memory impairment in BCAS mice during the probe phase (Figure S7K–P). Together, these behavioral data indicate that oligodendrocyte‐targeted *Sgk1.1* overexpression aggravates cognitive impairment in the BCAS model.

We next assessed white matter pathology. Black‐Gold and immunofluorescence staining revealed pronounced demyelination in the corpus callosum after BCAS, which was intensified by *Sgk1.1* overexpression (Figure S8A,B). Western blotting analyses likewise showed lower MBP and MAG protein abundance in the *Sgk1.1* overexpression group compared with BCAS controls (Figure S8C–E). Ultrastructural examination via transmission electron microscopy further supported these findings, showing thinner myelin sheaths in BCAS mice and higher g‐ratios upon *Sgk1.1* overexpression (Figure S8F,G).

Finally, we evaluated oligodendrocyte lineage status in the corpus callosum. While BCAS surgery led to a decline in the proportion of mature oligodendrocytes, *Sgk1.1* overexpression further reduced the fraction of mature oligodendrocytes (CC1^+^/Olig2^+^) compared with BCAS controls, without altering total Olig2^+^ cell density (Figure S9A–C). At the molecular level, quantitative PCR analyses additionally revealed that *Sgk1.1* overexpression suppressed the expression of myelin‐associated genes relative to BCAS mice (Figure S9D–G). Together, these gain‐of‐function findings indicate that elevated oligodendroglial *Sgk1.1* can exacerbate oligodendrocyte maturation deficits, white matter pathology, and cognitive impairment in the context of chronic cerebral hypoperfusion.

## 4 Discussion

In our study, by combining oligodendrocyte‐specific genetic knockdown and overexpression strategies with behavioral, pathological, and molecular detection, we demonstrate that an early, predominantly oligodendrocyte lineage‐associated increase in *Sgk1.1* mRNA occurs prior to overt WM injury. Oligodendrocyte lineage–targeted *Sgk1.1* knockdown alleviates maturation deficits of oligodendrocyte lineage cells, attenuates myelin injury, and ameliorates cognitive deficits in an AD model. Mechanistically, phosphoproteomic profiling coupled with site‐directed mutagenesis experiments identifies HDAC2 phosphorylation at S422/S424 as a functionally important downstream event associated with SGK1.1‐mediated suppression of oligodendrocyte maturation. In addition, validation in the BCAS model supports a broader role for SGK1.1 in promoting demyelination under chronic cerebral hypoperfusion. Collectively, our findings position oligodendrocyte‐associated *Sgk1.1* dysregulation as an early, functionally consequential factor associated with WM vulnerability and cognitive impairment in AD, and highlight the SGK1.1‐associated HDAC2 phospho‐regulatory axis as a potential therapeutic entry point to preserve WM integrity and mitigate cognitive decline.

AD has historically been conceptualized as a gray matter–dominant disorder driven primarily by neuronal and synaptic failure. However, converging neuropathological, neuroimaging, and single‐cell transcriptomic datasets increasingly implicate oligodendrocytes and myelin as active participants in disease evolution rather than passive bystanders [12, 24, 51, 52]. Neuroimaging studies using diffusion‐based metrics reveal that WM microstructural abnormalities are evident in MCI/early AD, correlate with cognitive decline, and may emerge before prominent cortical atrophy [53, 54, 55]. In parallel, transcriptomic analyses of human AD brains identify oligodendrocyte subtype–specific stress programs and altered differentiation states, suggesting disruption of myelin maintenance and lineage progression rather than a purely secondary consequence of neuronal loss [12, 51]. Experimental work further supports bidirectionality: myelin dysfunction can promote amyloid‐β deposition [11], whereas myelin renewal ameliorates cognitive impairment in AD mice [56], positioning myelin disruption as a disease‐amplifying and potentially tractable therapeutic target. Despite these advances, the drivers of AD‐associated WM injury remain poorly defined, particularly whether WM degeneration reflects primary disruptions in oligodendrocyte lineage progression and myelin maintenance programs. Most human transcriptomic atlases are derived from cross‐sectional postmortem cohorts and therefore capture associative relationships. As a result, they cannot establish the temporal ordering between oligodendrocyte‐lineage perturbations and WM pathology, nor can they identify the upstream cell‐intrinsic regulators that precipitate maturation arrest and consequent myelin decline in AD [12, 13, 57]. In this study, we sought to address these uncertainties by integrating age‐resolved temporal profiling with oligodendrocyte lineage–targeted genetic manipulation and multimodal detection of WM pathology and cognition. Through systematic assessment of myelin integrity across disease stages of 5 × FAD mice, we defined the period during which myelin disruption becomes detectable and identified an early increase in *Sgk1.1* mRNA that preceded overt WM injury and was predominantly detected in oligodendrocyte‐lineage cells. These observations provide a rationale for investigating SGK1.1 in AD‐associated WM injury.

Serum‐ and glucocorticoid‐regulated kinase 1 (SGK1) is a stress‐responsive AGC kinase that integrates signals from glucocorticoids, inflammatory mediators, and metabolic perturbations [58, 59, 60, 61]. Previous research has demonstrated its role in modulating neuronal excitability, synaptic function, and cellular stress responses within the central nervous system [35, 37, 62]. In the context of AD, SGK1 has been implicated in disease‐relevant neuronal dysfunction and tau‐associated pathology [40, 63]. Previous studies have also implicated SGK1 signaling in oligodendrocytes and myelin regulation [42, 44, 64]. However, the precise role of SGK1 in myelin damage associated with AD remains poorly understood, and the molecular mechanisms through which SGK1 alterations contribute to myelin dysfunction and neurodegeneration in AD have yet to be fully clarified. Notably, SGK1 comprises multiple isoforms. Among these, SGK1.1 is a brain‐specific splice isoform transcribed from an alternative promoter that shares the C‐terminal catalytic domain with canonical SGK1 but has a distinct N‐terminus that confers different stability and subcellular regulation [37, 45]. This distinction is particularly relevant for glial biology, as even subtle differences in molecular isoforms can exert disproportionate effects on glial cell function [65, 66]. In our study, we found that *Sgk1* mRNA remained largely stable from 4 to 6 months, whereas *Sgk1.1* mRNA increased at 5 months and remained elevated at 6 months, preceding overt myelin loss. Moreover, the disease‐associated increase in *Sgk1.1* mRNA was predominantly detected in Olig2^+^ cells. Functionally, oligodendrocyte lineage–targeted *Sgk1.1* knockdown alleviated maturation deficits, attenuated myelin injury, and improved cognitive performance in AD models. In contrast, overexpression of *Sgk1.1* was sufficient to suppress oligodendrocyte differentiation programs in vitro. Notably, consistent with prior work using the same MBP promoter‐driven chimeric AAV1/2 tool to target oligodendrocyte‐lineage cells across stages, we did not resolve potential stage‐dependent differences in SGK1.1 effects [48]. In addition, the lack of a validated isoform‐specific antibody precluded direct assessment of endogenous SGK1.1 protein changes, while cell‐resolved and functional comparisons between canonical SGK1 and SGK1.1 were not performed. These limitations constrain the stage‐ and isoform‐specific interpretation of our findings but do not alter our conclusion that oligodendrocyte‐associated *Sgk1.1* dysregulation contributes to WM vulnerability in 5 × FAD mice.

Oligodendrocyte differentiation and myelin formation require coordinated induction of myelin‐associated genes (e.g., *Mbp*, *Mag*, *Cnp*) and lineage‐defining transcriptional regulators such as MYRF [67, 68]. In particular, genetic studies have established HDAC1/2 as key gatekeepers of oligodendrocyte lineage progression that are essential for proper differentiation and myelin gene expression [31, 32]. Evidence from other biological systems has established that post‐translational modifications, and particularly phosphorylation, can dynamically tune HDAC activity by reshaping enzymatic function, interaction partners, and subcellular localization [33, 69, 70, 71], which provides a mechanistic precedent for the modulation of nuclear transcriptional capacity independently of changes in total protein abundance. Notably, S422/S424 have previously been implicated in basal HDAC2 phosphorylation and transcriptional repression [72, 73]. However, whether such phosphorylation‐dependent regulation of HDAC2 operates within oligodendrocytes remains largely unexplored. Given the kinase activity of SGK1.1, we employed unbiased phosphoproteomics to identify downstream signaling events. HDAC2 emerged as the only differentially phosphorylated candidate annotated to oligodendrocyte differentiation (GO:0048714). While *Sgk1.1* overexpression did not alter total HDAC2 protein abundance, it was associated with a significant increase in phosphorylation at Ser422/Ser424. Given the lack of validated phospho‐specific antibodies against S422/S424, HDAC2 phosphorylation was assessed by Phos‐tag SDS‐PAGE. The phosphorylated HDAC2 fraction was unchanged at 4 months, showed an upward trend at 5 months, and was significantly elevated at 6 months, broadly paralleling the age‐associated increase in *Sgk1.1* mRNA, while total HDAC2 levels remained stable. As Phos‐tag does not resolve individual phosphosites, the temporal dynamics of S422/S424 phosphorylation remain to be established. Notably, co‐immunoprecipitation further revealed an association between SGK1.1 and HDAC2, supporting a functional link between SGK1.1 and HDAC2. Crucially, subsequent phospho‐site mutagenesis demonstrated the functional importance of these sites. The phosphomimetic mutant (S422D/S424D) was sufficient to mimic the SGK1.1‐induced suppression of myelin‐associated genes and proteins, whereas the phospho‐deficient mutant (S422A/S424A) antagonized this effect. Together, these findings support a functional role for HDAC2 S422/S424 phosphorylation in SGK1.1‐associated suppression of oligodendrocyte maturation. However, because a cell‐free kinase assay was not performed, an indirect mechanism involving an intermediate kinase cannot be excluded. The structural basis of this regulation remains to be fully elucidated. Specifically, it is not yet known whether these functional outputs are mediated by the unique N‐terminal domain of SGK1.1 or the C‐terminal catalytic domain shared with canonical SGK1. Resolving these structural determinants and the precise subcellular localization of the SGK1.1‐HDAC2 association will be a focus of our future investigations.

Chronic cerebral hypoperfusion is a major contributor to age‐related cognitive impairment and frequently coexists with AD pathology [74, 75, 76]. To study this, the bilateral common carotid artery stenosis (BCAS) model serves as a standard paradigm, recapitulating white matter–predominant injury and cognitive deficits driven by sustained reductions in cerebral blood flow [77, 78]. In contrast to AD‐associated demyelination, which emerges from a multifactorial and complex pathological landscape, BCAS‐evoked white matter damage is directly induced by experimentally controlled hemodynamic deficit [78]. This etiologic difference may underlie the opposite trends in *Sgk1.1* mRNA expression observed across the two models. Although the biological significance of the endogenous *Sgk1.1* decrease after BCAS remains unclear, forced *Sgk1.1* overexpression significantly exacerbated demyelination and cognitive deficits. These gain‐of‐function findings indicate that elevated oligodendroglial *Sgk1.1* can exacerbate white matter injury in an independent pathological context, complementing the loss‐of‐function evidence obtained in the 5 × FAD mice. Notably, across both AD and BCAS, canonical *Sgk1* mRNA remained largely unchanged, whereas *Sgk1.1* showed context‐dependent regulation, underscoring the importance of resolving SGK1 isoforms separately when examining white matter pathology.

Beyond the mechanistic caveats discussed above, the scope and generalizability of these findings remain limited by several aspects of the experimental design. Our temporal analyses in 5 × FAD mice were confined to 4–6 months of age. Given the progressive deterioration of myelin and oligodendrocyte progenitor function with aging, together with the continued evolution of the 5 × FAD phenotype at later ages, whether *Sgk1.1* dysregulation and the beneficial effects of its knockdown persist at advanced disease stages remain to be determined [79, 80, 81]. Furthermore, all in vivo experiments were conducted in male mice, and documented sex‐related differences in AD pathological burden and genetic risk warrant validation of these findings in females [82, 83]. Finally, whether *Sgk1.1* transcript abundance and HDAC2 S422/S424 phosphorylation show corresponding alterations in human AD white matter remains to be established in well‐characterized postmortem cohorts.

In summary, our study identifies oligodendrocyte‐associated *Sgk1.1* dysregulation as a functionally relevant contributor to early oligodendrocyte maturation deficits and impaired myelin integrity in AD. Mechanistically, our data link SGK1.1 signaling to HDAC2 S422/S424 phosphorylation, supporting a mechanistic pathway through which SGK1.1 may impair oligodendrocyte differentiation programs. The BCAS validation further supports the relevance of SGK1.1 to white matter vulnerability across distinct etiologic contexts. Collectively, these findings highlight SGK1.1‐targeted intervention as a potential therapeutic strategy for protecting myelin and sustaining cognitive function in AD and related white matter disorders.

## 5 Conclusions

Our findings identify oligodendrocyte‐associated *Sgk1.1* mRNA elevation as an early event preceding WM pathology in AD models and reveal a functional link between SGK1.1 signaling and HDAC2 S422/S424 phosphorylation that contributes to impaired oligodendrocyte maturation and myelin gene expression. Targeting the SGK1.1–HDAC2 phospho‐axis may represent a therapeutic strategy to support myelin maintenance and cognitive function in AD and associated demyelinating pathologies.

## Author Contributions

Shiji Deng, Tingting Wang, Yun Xu, and Xiaolei Zhu initiated and designed the research. Shiji Deng and Xiaolei Zhu wrote the manuscript. Shiji Deng, Shenghan Gao, Zhen Lan, Chao Zhou, Huiya Li, and Jiang Chen performed all the experiments and analyzed the results. Jiashuo Li, Siyuan Zhang, Xinyu Bao, Yuhao Xu, and Shenghan Gao helped to collect the results. Pinyi Liu contributed to the discussion of the results. Xiaolei Zhu supervised the entire project. All authors contributed to and have approved the final manuscript.

## Funding

This work was supported by the National Natural Science Foundation of China (823B2027), the STI2030‐Major Projects (2022ZD0211800), and Jiangsu Province Key Medical Discipline (ZDXK202216).

## Ethics Statement

All animal procedures were reviewed and approved by the Institutional Animal Care and Use Committee of Nanjing University (2025AE01031).

## Consent

The authors have nothing to report.

## Conflicts of Interest

The authors declare that they have no competing interests. Yun Xu serves as an Editorial Board Member of CNS Neuroscience and Therapeutics and is also a co‐author of this manuscript. In order to mitigate potential bias, Yun Xu was not involved in any editorial decision‐making processes related to the acceptance of this article for publication.

## Supporting information


**Figure S1:** cns71150‐sup‐0001‐FigureS1‐S9‐TableS1.docx. *Sgk1.1* mRNA is elevated in Olig2^+^ cells within the corpus callosum of 5 × FAD mice at 5 and 6 months of age.
(A) Representative RNAscope images of Sgk1.1 mRNA combined with Olig2 immunofluorescence and DAPI counterstaining in the corpus callosum of 4‐, 5‐, and 6‐month‐old 5 × FAD mice and age‐matched littermate WT controls. Enlarged views show selected Olig2+ cells with detectable *Sgk1.1* signal and corresponding DAPI images. Scale bars, 17 μm (overview) and 1.7 μm (enlarged view).
(B, C) Quantification of the relative *Sgk1.1*‐positive area per Olig2⁺ cell (B) or Olig2⁻ cell (C) in age‐matched WT and 5 × FAD mice. Values were normalized to the mean value of Olig2⁺ cells from 4‐month‐old WT mice. *n* = 4 mice per group.
(D, E) Quantification of the relative *Sgk1.1*‐positive area per Olig2⁺ or Olig2⁻ cell across 4, 5, and 6 months of age in WT mice (D) and 5 × FAD mice (E). Values were normalized to the mean value of Olig2⁺ cells from 4‐month‐old WT mice. *n* = 4 mice per group.
Data are presented as mean ± SEM. *p*‐values were determined by two‐tailed Student's *t*‐test in (B, C). Comparisons across ages in D, E were performed separately within the Olig2⁺ and Olig2⁻ compartments using one‐way ANOVA with Tukey's post hoc analysis. ns, not significant; *p < 0.05, **p < 0.01, ***p < 0.001.
**Figure S2:** Oligodendrocyte‐targeted Sgk1.1 knockdown reduces Olig2‐associated Sgk1.1 mRNA signal in the cortex and corpus callosum of 5 × FAD mice.
(A) Representative RNAscope images of Sgk1.1 mRNA combined with Olig2 immunofluorescence and DAPI counterstaining in the cortex and corpus callosum of 6‐month‐old 5 × FAD‐GFP and 5 × FAD‐KD mice. Enlarged views show selected Olig2+ cells with detectable *Sgk1.1* signal and corresponding DAPI images. Scale bars, 17 μm (overview) and 1.7 μm (enlarged view). (B, C) Quantification of the relative Sgk1.1‐positive area per Olig2+ or Olig2⁻ cell in the cortex (B) and corpus callosum (C). Values were normalized to the mean value of the corresponding 5 × FAD‐GFP group. *n* = 4 mice per group.
Data are presented as mean ± SEM. *p*‐values were determined by two‐tailed Student's *t*‐test in B, C. ns, not significant; **p < 0.01.
**Figure S3:** Oligodendrocyte‐targeted knockdown of Sgk1.1 does not significantly alter total oligodendrocyte‐lineage cell density in 6‐month‐old 5 × FAD mice. (A, D, G) Representative immunofluorescence images of PDGFRα and Olig2 in the cortex (A), corpus callosum (D), and hippocampus (G) of 6‐month‐old WT‐GFP, 5 × FAD‐GFP, and 5 × FAD‐KD mice. Scale bar, 50 μm. (B, E, H) Quantification of total Olig2⁺ cell density in the cortex (B), corpus callosum (E), and hippocampus (H). *n* = 4 mice per group. (C, F, I) Quantification of PDGFRα⁺/Olig2⁺ OPC density in the cortex (C), corpus callosum (F), and hippocampus (I). *n* = 4 mice per group. Data are presented as mean ± SEM. *p*‐values were determined by one‐way ANOVA with Tukey's post hoc analysis in B, C, E, F, and H, I. ns, not significant; *p < 0.05, **p < 0.01.
**Figure S4:** Overexpression of Sgk1.1 suppresses oligodendrocyte maturation in vitro. (A) Schematic illustration of the experimental workflow, including OPC isolation, proliferation, differentiation, and viral transduction. (B) Representative immunofluorescence images showing viral transduction (GFP) in cultured oligodendrocyte‐lineage cells. Scale bar, 50 μm. (C, D) Quantitative PCR analysis of Sgk1 (C) and Sgk1.1 (D) mRNA expression in oligodendrocytes transduced with control virus (OL‐GFP) or Sgk1.1 overexpression virus (OL‐OE). Gene expression was normalized to Actb and then normalized to the mean value of the OL‐GFP group. *n* = 3 per group. (E, F) Representative immunoblot bands (E) and quantification (F) of MAG and MBP protein levels in OL‐GFP and OL‐OE cells. Band intensities were normalized to β‐actin and then normalized to the mean value of the OL‐GFP group. *n* = 4 per group. (G–J) Quantitative PCR analysis of *Myrf* (G), *Mag* (H), *Mbp* (I), and *Cnp* (J) mRNA expression in OL‐GFP and OL‐OE cells. Gene expression was normalized to Actb and then normalized to the mean value of the OL‐GFP group. *n* = 3–4 per group. Data are presented as mean ± SEM. *p*‐values were determined by two‐tailed Student's *t*‐test in C–D and F–J. ns, not significant; *p < 0.05, **p < 0.01, ***p < 0.001.
**Figure S5:** Temporal changes in HDAC2 phosphorylation in the corpus callosum of 4‐ to 6‐month‐old 5 × FAD mice. (A) Representative Phos‐tag immunoblot bands of HDAC2 in corpus callosum lysates from 4‐, 5‐, and 6‐month‐old 5 × FAD mice and age‐matched WT controls. β‐actin served as a loading control. (B–D) Quantification of total HDAC2 protein levels at 4 months (B), 5 months (C), and 6 months (D). Band intensities were normalized to β‐actin and then normalized to the mean value of the corresponding WT group. *n* = 3 mice per group. (E–G) Quantification of the phosphorylated fraction of HDAC2 at 4 months (E), 5 months (F), and 6 months (G). The phosphorylated fraction was calculated as the intensity of the slower‐migrating phosphorylated HDAC2 signal divided by the total HDAC2 signal in the same lane and then normalized to the mean value of the corresponding WT group. *n* = 3 mice per group. Data are presented as mean ± SEM. *p*‐values were determined by two‐tailed Student's *t*‐test in B–G. ns, not significant; *p < 0.05.
**Figure S6:** Association between SGK1.1 and HDAC2 in primary oligodendrocyte‐lineage cells. (A–B) Two additional independent Co‐IP experiments examining the association between SGK1.1‐FLAG and HDAC2 in primary oligodendrocyte‐lineage cells.
(C) Quantification of the HDAC2/FLAG ratio in FLAG immunoprecipitates across three independent Co‐IP experiments, including Figure 6G and A, B. *n* = 3 independent experiments.
**Figure S7:** Oligodendrocyte‐targeted Sgk1.1 overexpression exacerbates cognitive impairment in BCAS mice. (A) Schematic diagram of the BCAS surgery and sample collection procedure.
(B–C) Quantitative PCR analysis of *Sgk1* (B) and *Sgk1.1* (C) mRNA expression in the corpus callosum of Sham and BCAS mice. Gene expression was normalized to *Actb* and then normalized to the mean value of the Sham group. *n* = 3 mice per group.
(D) Schematic diagram of Sgk1.1 overexpression, BCAS surgery, and subsequent behavioral tests and tissue collection for analysis. (E–F) Representative images of viral transduction in the corpus callosum (E) and co‐localization with Olig2 (F). Scale bars, 200 μm (E) and 50 μm (F). (G–H) Quantitative PCR analysis of Sgk1 (G) and Sgk1.1 (H) mRNA expression in the corpus callosum of BCAS mice injected with Sgk1.1‐overexpression virus (BCAS‐OE) or control virus (BCAS‐GFP). Gene expression was normalized to Actb and then normalized to the mean value of the BCAS‐GFP group. *n* = 4 mice per group. (I) Open field test showing total distance traveled, time spent in corner zones, and time spent in the central zone for Sham‐GFP, BCAS‐GFP, and BCAS‐OE mice. *n* = 12 mice per group. (J) Y‐maze spontaneous alternation (%) in Sham‐GFP, BCAS‐GFP, and BCAS‐OE mice. *n* = 12 mice per group. (K–P) Morris water maze performance of Sham‐GFP, BCAS‐GFP, and BCAS‐OE mice, including escape latency over the first five days (K), swimming speed (L), latency to first entry into the target quadrant during the probe trial (M), platform crossing times during the probe trial (N), time spent in the target quadrant (O), and representative swimming paths (P). *n* = 12 mice per group. Data are presented as mean ± SEM. *p*‐values were determined by two‐tailed Student's *t*‐test in B, C and G‐H, by one‐way ANOVA with Tukey's post hoc analysis in I–J, the Day 5 escape latency in K, L, and N, O, and by Kruskal–Wallis test followed by Dunn's post hoc test in M. ns, not significant, *p < 0.05, **p < 0.01.
**Figure S8:** Oligodendrocyte‐targeted Sgk1.1 overexpression exacerbates demyelination in BCAS mice. (A) Representative Black Gold staining images and immunofluorescence images of MAG and MBP in the corpus callosum of Sham‐GFP, BCAS‐GFP, and BCAS‐OE mice. Scale bar, 200 μm. (B) Quantification of the positive area (%) of Black Gold staining and MAG and MBP immunofluorescence in the corpus callosum of Sham‐GFP, BCAS‐GFP, and BCAS‐OE mice. *n* = 4 mice per group. (C) Representative immunoblot bands of MBP and MAG in the corpus callosum of Sham‐GFP, BCAS‐GFP, and BCAS‐OE mice. (D–E) Quantification of MAG (D) and MBP (E) protein levels in the corpus callosum of Sham‐GFP, BCAS‐GFP, and BCAS‐OE mice. Band intensities were normalized to β‐actin and then normalized to the mean value of the Sham‐GFP group. *n* = 4 mice per group. (F) Representative TEM images of myelinated axons in the corpus callosum of Sham‐GFP, BCAS‐GFP, and BCAS‐OE mice. Scale bar, 2 μm. (G) Quantification of g‐ratio in the indicated experimental groups, grouped by axon diameter. *n* = 3 mice per group. Data are presented as mean ± SEM. *p*‐values were determined by one‐way ANOVA with Tukey's post hoc analysis in B, D, E, and G. *p < 0.05, **p < 0.01, ***p < 0.001.
**Figure S9:** Oligodendrocyte‐targeted Sgk1.1 overexpression further impairs oligodendrocyte maturation in BCAS mice. (A) Representative immunofluorescence images of Olig2 and CC1 co‐staining in the corpus callosum of Sham‐GFP, BCAS‐GFP, and BCAS‐OE mice. Scale bar, 50 μm. (B) Quantification of oligodendrocyte maturation in the corpus callosum, expressed as the percentage of CC1⁺ cells among Olig2⁺ cells (CC1⁺/Olig2⁺). *n* = 4 mice per group. (C) Quantification of Olig2⁺ cell density in the corpus callosum of Sham‐GFP, BCAS‐GFP, and BCAS‐OE mice. *n* = 4 mice per group. (D–G) Quantitative PCR analysis of *Myrf* (D), *Mag* (E), *Mbp* (F), and *Cnp* (G) mRNA expression in the corpus callosum of BCAS‐GFP and BCAS‐OE mice. Gene expression was normalized to Actb and then normalized to the mean value of the BCAS‐GFP group. *n* = 3–4 mice per group. Data are presented as mean ± SEM. *p*‐values were determined by one‐way ANOVA with Tukey's post hoc analysis in B‐C, and by two‐tailed Student's *t*‐test in D–G. ns, not significant, *p < 0.05, **p < 0.01, ***p < 0.001.

**Table S1:** Differentially upregulated phosphosites identified by phosphoproteomic profiling upon *Sgk1.1* overexpression. The additional file shows this in more detail. Bold text indicates candidate phosphorylation sites on HDAC2.

## Data Availability

The phosphoproteomics data reported in this paper have been deposited in the OMIX, China National Center for Bioinformation/Beijing Institute of Genomics, Chinese Academy of Sciences (https://ngdc.cncb.ac.cn/omix: accession no. OMIX014540) and will be publicly available upon publication of this article. Other data that support the findings of this study are available from the corresponding author upon reasonable request (Xiaolei Zhu, zhuquelee@126.com).
