# Supplementary material for: Oligodendrocyte‐Targeted SGK1.1 Inhibition Alleviates White Matter Injury and Cognitive Impairment in Alzheimer's Disease via Modulation of HDAC2 Phosphorylation
Source: CNS Neurosci Ther. 2026 Sep 8;32(9):e71150. doi: 10.1002/cns.71150 (PMC13554428; doi:10.1002/cns.71150)
Supplement: Supplementary file 1 [file CNS-32-e71150-s001.docx]

**Oligodendrocyte-Targeted SGK1.1 Inhibition Alleviates White Matter Injury and Cognitive Impairment in Alzheimer’s Disease via Modulation of HDAC2 Phosphorylation**

Shiji Deng, Shenghan Gao, Chao Zhou, Zhen Lan, Huiya Li, Jiang Chen, Yuhao Xu, Jiashuo Li, Pinyi Liu, Siyuan Zhang, Xinyu Bao, Tingting Wang, Yun Xu, Xiaolei Zhu

**Include:**

Figures S1-S9

Table S1

**Figure S1. *Sgk1.1* mRNA is elevated in Olig2^+^ cells within the corpus callosum of 5×FAD mice at 5 and 6 months of age.**

(A) Representative RNAscope images of *Sgk1.1* mRNA combined with Olig2 immunofluorescence and DAPI counterstaining in the corpus callosum of 4-, 5-, and 6-month-old 5×FAD mice and age-matched littermate WT controls. Enlarged views show selected Olig2^+^ cells with detectable *Sgk1.1* signal and corresponding DAPI images. Scale bars, 17 μm (overview) and 1.7 μm (enlarged view).

(B–C) Quantification of the relative *Sgk1.1*-positive area per Olig2⁺ cell (B) or Olig2⁻ cell (C) in age-matched WT and 5×FAD mice. Values were normalized to the mean value of Olig2⁺ cells from 4-month-old WT mice. n = 4 mice per group.

(D–E) Quantification of the relative *Sgk1.1*-positive area per Olig2⁺ or Olig2⁻ cell across 4, 5, and 6 months of age in WT mice (D) and 5×FAD mice (E). Values were normalized to the mean value of Olig2⁺ cells from 4-month-old WT mice. n = 4 mice per group.

Data are presented as mean ± SEM. p-values were determined by two-tailed Student’s *t*-test in B–C. Comparisons across ages in D–E were performed separately within the Olig2⁺ and Olig2⁻ compartments using one-way ANOVA with Tukey’s post hoc analysis. ns, not significant; **p* < 0.05, ***p* < 0.01, ****p* < 0.001.

**Figure S2. Oligodendrocyte-targeted *Sgk1.1* knockdown reduces Olig2-associated *Sgk1.1* mRNA signal in the cortex and corpus callosum of 5×FAD mice.**

(A) Representative RNAscope images of *Sgk1.1* mRNA combined with Olig2 immunofluorescence and DAPI counterstaining in the cortex and corpus callosum of 6-month-old 5×FAD-GFP and 5×FAD-KD mice. Enlarged views show selected Olig2^+^ cells with detectable *Sgk1.1* signal and corresponding DAPI images. Scale bars, 17 μm (overview) and 1.7 μm (enlarged view).

(B–C) Quantification of the relative *Sgk1.1*-positive area per Olig2^+^ or Olig2⁻ cell in the cortex (B) and corpus callosum (C). Values were normalized to the mean value of the corresponding 5×FAD-GFP group. n = 4 mice per group.

Data are presented as mean ± SEM. p-values were determined by two-tailed Student’s *t*-test in B–C. ns, not significant; ***p* < 0.01.

**Figure S3. Oligodendrocyte-targeted knockdown of *Sgk1.1* does not significantly alter total oligodendrocyte-lineage cell density in 6-month-old 5×FAD mice.**

(A, D, G) Representative immunofluorescence images of PDGFRα and Olig2 in the cortex (A), corpus callosum (D), and hippocampus (G) of 6-month-old WT-GFP, 5×FAD-GFP, and 5×FAD-KD mice. Scale bar, 50 μm.

(B, E, H) Quantification of total Olig2⁺ cell density in the cortex (B), corpus callosum (E), and hippocampus (H). n = 4 mice per group.

(C, F, I) Quantification of PDGFRα⁺/Olig2⁺ OPC density in the cortex (C), corpus callosum (F), and hippocampus (I). n = 4 mice per group.

Data are presented as mean ± SEM. p-values were determined by one-way ANOVA with Tukey’s post hoc analysis in B–C, E–F, and H–I. ns, not significant; *p < 0.05, **p < 0.01.

**Figure S4. Overexpression of *Sgk1.1* suppresses oligodendrocyte maturation *in vitro.***

(A) Schematic illustration of the experimental workflow, including OPC isolation, proliferation, differentiation, and viral transduction.

(B) Representative immunofluorescence images showing viral transduction (GFP) in cultured oligodendrocyte-lineage cells. Scale bar, 50 μm.

(C–D) Quantitative PCR analysis of Sgk1 (C) and Sgk1.1 (D) mRNA expression in oligodendrocytes transduced with control virus (OL-GFP) or *Sgk1.1* overexpression virus (OL-OE). Gene expression was normalized to Actb and then normalized to the mean value of the OL-GFP group. n = 3 per group.

(E–F) Representative immunoblot bands (E) and quantification (F) of MAG and MBP protein levels in OL-GFP and OL-OE cells. Band intensities were normalized to β-actin and then normalized to the mean value of the OL-GFP group. n = 4 per group.

(G–J) Quantitative PCR analysis of Myrf (G), Mag (H), Mbp (I), and Cnp (J) mRNA expression in OL-GFP and OL-OE cells. Gene expression was normalized to Actb and then normalized to the mean value of the OL-GFP group. n = 3–4 per group.

Data are presented as mean ± SEM. *p*-values were determined by two-tailed Student’s t-test in C–D and F–J. ns, not significant; **p* < 0.05, ***p* < 0.01,****p* < 0.001.

**Figure S5. Temporal changes in HDAC2 phosphorylation in the corpus callosum of 4- to 6-month-old 5×FAD mice.**

(A) Representative Phos-tag immunoblot bands of HDAC2 in corpus callosum lysates from 4-, 5-, and 6-month-old 5×FAD mice and age-matched WT controls. β-actin served as a loading control.

(B–D) Quantification of total HDAC2 protein levels at 4 months (B), 5 months (C), and 6 months (D). Band intensities were normalized to β-actin and then normalized to the mean value of the corresponding WT group. n = 3 mice per group.

(E–G) Quantification of the phosphorylated fraction of HDAC2 at 4 months (E), 5 months (F), and 6 months (G). The phosphorylated fraction was calculated as the intensity of the slower-migrating phosphorylated HDAC2 signal divided by the total HDAC2 signal in the same lane and then normalized to the mean value of the corresponding WT group. n = 3 mice per group.

Data are presented as mean ± SEM. p-values were determined by two-tailed Student’s *t*-test in B–G. ns, not significant; **p* < 0.05.

**Figure S6. Association between SGK1.1 and HDAC2 in primary** **oligodendrocyte-lineage cells.**

(A–B) Two additional independent Co-IP experiments examining the association between SGK1.1-FLAG and HDAC2 in primary oligodendrocyte-lineage cells.

(C) Quantification of the HDAC2/FLAG ratio in FLAG immunoprecipitates across three independent Co-IP experiments, including Fig. 6G and A–B. n = 3 independent experiments.

**Figure S7. Oligodendrocyte-targeted *Sgk1.1* overexpression exacerbates cognitive impairment in BCAS mice.**

(A) Schematic diagram of the BCAS surgery and sample collection procedure.

(B–C) Quantitative PCR analysis of Sgk1 (B) and Sgk1.1 (C) mRNA expression in the corpus callosum of Sham and BCAS mice. Gene expression was normalized to Actb and then normalized to the mean value of the Sham group. n = 3 mice per group.

(D) Schematic diagram of *Sgk1.1* overexpression, BCAS surgery, and subsequent behavioral tests and tissue collection for analysis.

(E–F) Representative images of viral transduction in the corpus callosum (E) and co-localization with Olig2 (F). Scale bars, 200 μm (E) and 50 μm (F).

(G–H) quantitative PCR analysis of Sgk1 (G) and Sgk1.1 (H) mRNA expression in the corpus callosum of BCAS mice injected with *Sgk1.1*-overexpression virus (BCAS-OE) or control virus (BCAS-GFP). Gene expression was normalized to *Actb* and then normalized to the mean value of the BCAS-GFP group. n = 4 mice per group.

(I) Open field test showing total distance traveled, time spent in corner zones, and time spent in the central zone for Sham-GFP, BCAS-GFP, and BCAS-OE mice. n = 12 mice per group.

(J) Y-maze spontaneous alternation (%) in Sham-GFP, BCAS-GFP, and BCAS-OE mice. n = 12 mice per group.

(K–P) Morris water maze performance of Sham-GFP, BCAS-GFP, and BCAS-OE mice, including escape latency over the first five days (K), swimming speed (L), latency to first entry into the target quadrant during the probe trial (M), platform crossing times during the probe trial (N), time spent in the target quadrant (O), and representative swimming paths (P). n = 12 mice per group.

**Data are presented as mean ± SEM. *p*-values were determined by two-tailed Student's** t**-test in B–C and G-H, by one-way ANOVA with Tukey’s post hoc analysis in I–J, the day5 escape latency in K,L, and N–O, and by** Kruskal–Wallis test followed by Dunn’s post hoc test in M. ns, not significant, **p* < 0.05, ***p* < 0.01.

**Figure S8. Oligodendrocyte-targeted *Sgk1.1* overexpression exacerbates demyelination in BCAS mice.**

(A) Representative Black Gold staining images and immunofluorescence images of MAG and MBP in the corpus callosum of Sham-GFP, BCAS-GFP, and BCAS-OE mice. Scale bar, 200 μm.

(B) Quantification of the positive area (%) of Black Gold staining and MAG and MBP immunofluorescence in the corpus callosum of Sham-GFP, BCAS-GFP, and BCAS-OE mice. n = 4 mice per group.

(C) Representative immunoblot bands of MBP and MAG in the corpus callosum of Sham-GFP, BCAS-GFP, and BCAS-OE mice.

(D–E) Quantification of MAG (D) and MBP (E) protein levels in the corpus callosum of Sham-GFP, BCAS-GFP, and BCAS-OE mice. Band intensities were normalized to β-actin and then normalized to the mean value of the Sham-GFP group. n = 4 mice per group.

(F) Representative TEM images of myelinated axons in the corpus callosum of Sham-GFP, BCAS-GFP, and BCAS-OE mice. Scale bar, 2 μm.

(G) Quantification of g-ratio in the indicated experimental groups, grouped by axon diameter. n = 3 mice per group. Data are presented as mean ± SEM. *p*-values were determined by one-way ANOVA with Tukey’s post hoc analysis in B, D–E, and G. **p* < 0.05, ***p* < 0.01,****p* < 0.001.

**Figure S9. Oligodendrocyte-targeted *Sgk1.1* overexpression further impairs oligodendrocyte maturation in BCAS mice.**

(A) Representative immunofluorescence images of Olig2 and CC1 co-staining in the corpus callosum of Sham-GFP, BCAS-GFP, and BCAS-OE mice. Scale bar, 50 μm.

(B) Quantification of oligodendrocyte maturation in the corpus callosum, expressed as the percentage of CC1⁺ cells among Olig2⁺ cells (CC1⁺/Olig2⁺). n = 4 mice per group.

(C) Quantification of Olig2⁺ cell density in the corpus callosum of Sham-GFP, BCAS-GFP, and BCAS-OE mice. n = 4 mice per group.

(D–G) Quantitative PCR analysis of Myrf (D), Mag (E), Mbp (F), and Cnp (G) mRNA expression in the corpus callosum of BCAS-GFP and BCAS-OE mice. Gene expression was normalized to Actb and then normalized to the mean value of the BCAS-GFP group. n = 3–4 mice per group.

**Data are presented as mean ± SEM. *p*-values were determined by one-way ANOVA with Tukey’s post hoc analysis in B-C, and by two-tailed Student's** t**-test in D–G.** ns, not significant, **p* < 0.05, ***p* < 0.01,****p* < 0.001.

**Table S1. Differentially upregulated phosphosites identified by phosphoproteomic profiling upon *Sgk1.1* overexpression.**

The additional file shows this in more detail. Bold text indicates candidate phosphorylation sites on HDAC2.
